# Surface Modification of Graphene Oxide for Fast Removal of Per- and Polyfluoroalkyl Substances (PFAS) Mixtures from River Water

**DOI:** 10.1021/acsestwater.4c00187

**Published:** 2024-06-11

**Authors:** Md. Nahid Pervez, Tao Jiang, Jaydev Kumar Mahato, Aswin Kumar Ilango, Yamini Kumaran, Yuwei Zuo, Weilan Zhang, Haralabos Efstathiadis, Jeremy I. Feldblyum, Mehmet V. Yigit, Yanna Liang

**Affiliations:** † Department of Environmental and Sustainable Engineering, University at Albany, State University of New York, Albany, New York 12222, United States; ‡ Department of Nanoscale Science and Engineering, University at Albany, State University of New York, Albany, New York 12222, United States; § Department of Chemistry, University at Albany, State University of New York, Albany, New York 12222, United States

**Keywords:** per- and polyfluoroalkyl substances (PFAS), cationic graphene oxide, surfactant, fast adsorption kinetics, river water

## Abstract

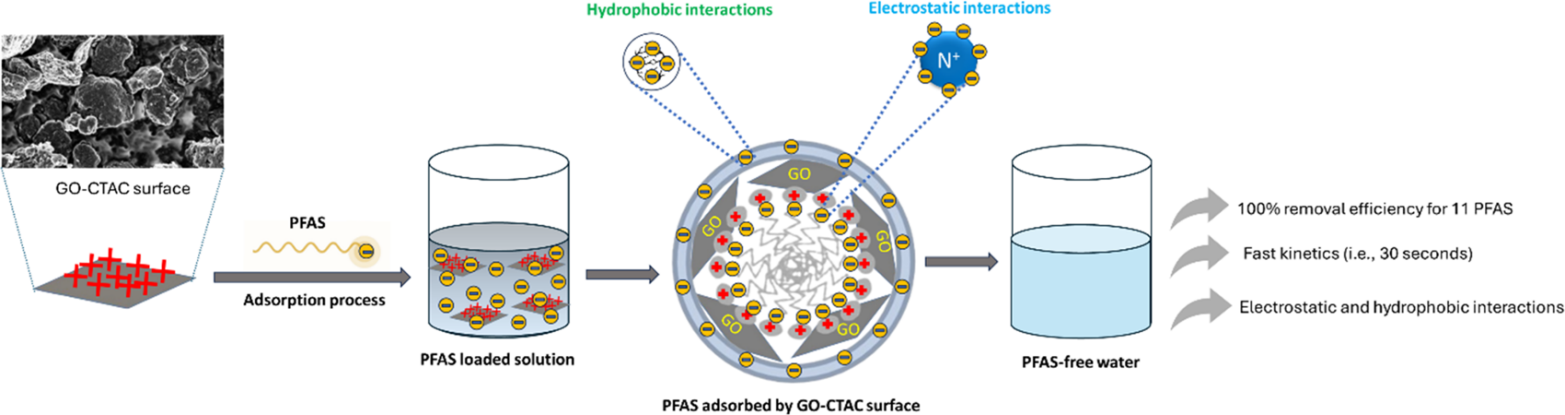

Per- and polyfluoroalkyl substances (PFAS) make up a diverse group of industrially derived organic chemicals that are of significant concern due to their detrimental effects on human health and ecosystems. Although other technologies are available for removing PFAS, adsorption remains a viable and effective method. Accordingly, the current study reported a novel type of graphene oxide (GO)-based adsorbent and tested their removal performance toward removing PFAS from water. Among the eight adsorbents tested, GO modified by a cationic surfactant, cetyltrimethylammonium chloride (CTAC), GO-CTAC was found to be the best, showing an almost 100% removal for all 11 PFAS tested. The adsorption kinetics were best described by the pseudo-second-order model, indicating rapid adsorption. The isotherm data were well supported by the Toth model, suggesting that PFAS adsorption onto GO-CTAC involved complex interactions. Detailed characterization using scanning electron microscopy-energy dispersive X-ray spectroscopy, Fourier transform infrared, thermogravimetric analysis, X-ray diffraction, and X-ray photoelectron spectroscopy confirmed the proposed adsorption mechanisms, including electrostatic and hydrophobic interactions. Interestingly, the performance of GO-CTAC was not influenced by the solution pH, ionic strength, or natural organic matter. Furthermore, the removal efficiency of PFAS at almost 100% in river water demonstrated that GO-CTAC could be a suitable adsorbent for capturing PFAS in real surface water.

## Abstract 2

Graphene oxide coated with cetyltrimethylammonium chloride rapidly removed a mixture of short- and long-chain PFAS in river water.

## 1 Introduction

Due to their robust chemical and thermal properties, per- and polyfluoroalkyl substances (PFAS) have been utilized in numerous applications such as coatings, textiles, adhesives, cosmetics, food packaging, and aqueous film-forming foams.^1,2^ PFAS are characterized by the presence of both a polar head and a hydrophobic tail that contain high energy C–F bonds (544 kJ/mol) within their molecular structures.^3^ This molecular framework contributes to PFAS’ high water solubility and exceptional environmental persistence, leading to their classification as “forever chemicals”. In addition, PFAS have been reported to be highly toxic to human beings, plants, and animals.^4−6^ Given all of the concerns tied to PFAS, environmental protection agencies at both the state and federal levels in the U.S. have established a series of legal standards (i.e., maximum contaminant levels) and health advisory levels (HAL). For example, the lifetime HAL by the U.S. Environmental Protection Agency (EPA) in 2022 for PFAS in drinking water stipulates that the concentration of perfluorooctanoic acid (PFOA) and perfluorooctanesulfonate (PFOS) should not exceed 0.004 and 0.02 ng/L, respectively.^7^ Hence, technologies that can completely remove PFAS or decrease PFAS concentrations in drinking water to such extremely low levels are urgently needed.

Various approaches have been explored for the treatment of PFAS, such as adsorption, membrane filtration, chemical/electrochemical destruction, and biological degradation.^8,9^ Among them, the adsorption technique is well recognized as a cost-effective and efficient method for quickly removing PFAS from polluted water sources.^10,11^ Traditional adsorbents, such as granular activated carbon (GAC), have been used to capture PFAS in water treatment facilities and residential point-of-use systems. Nevertheless, these adsorbents encounter drawbacks, such as slow adsorption rates, no selectivity toward PFAS, and weak affinity toward short-chain and relatively hydrophilic PFAS.^12−14^ For example, the adsorption of four-carbon perfluorobutanoic acid (PFBA) by GAC was 5 to >10 times less than that of eight-carbon PFOA in both batch and column trials.^15^ Eschauzier et al. also reported that using GAC was not successful in removing PFBA in real-world scenarios compared to PFOA or PFOS.^16^ Therefore, adsorbents that have faster adsorption rates and larger adsorption capacities than GAC for all short- and long-chain PFAS are highly desired.

In recent years, graphene oxide (GO) has received significant interest as a material for treating wastewater effluent due to its high surface area and abundance of oxygen-containing functional groups, including hydroxyl, carboxyl, and epoxy groups.^17,18^ Recently, GO-based materials have shown considerable promise in removing PFAS from water.^19,20^ Nevertheless, GO’s ability to adsorb anionic PFAS is restricted by its negatively charged surface and hydrophilic nature. Therefore, to use GO as an adsorbent for PFAS, structure modification is necessary. For example, Lei et al.^21^ enhanced the adsorption of PFOA in water by modifying GO with cationic polymer polyethylenimine (PEI), resulting in an adsorption capacity of about 368.2 mg/g. Similarly, Tian et al.^22^ modified GO by including ethylene diamine, leading to an adsorption capacity of 1575 mg/g for PFOA.^23,24^ Despite these successes, these investigations have identified several concerns, such as the inadequate stability of GO, the slow rate of PFAS adsorption, and the limited number of PFAS studied.^25,26^ In light of the fact that multiple PFAS are often detected in contaminated environments, adsorbents that are able to adsorb all forms of PFAS at a fast rate are needed and preferred.

Interestingly, surfactant-modified GO exhibits enhanced dispersibility and stability in both aqueous and organic solvents. The extent and manner in which surfactants and GO interact mostly rely on the concentration and characteristics of the surfactant.^27^ So far, several researchers have endeavored to enhance the properties of GO by including suitable surfactants for wastewater treatment. For example, Kuang et al.^28^ documented loading hexadecyltrimethylammonium bromide (HDTMA), a cationic surfactant, onto a GO-based adsorbent. The newly synthesized adsorbent effectively removed copper ions and bisphenol A from the water. The adsorption kinetics for Cu^2+^ and bisphenol A were fast, achieving almost 100% removal within 1 and 2 h, respectively. A separate investigation showed that the surfactant/GO composite had an adsorption capacity of 15 times more than the original GO.^29^ This indicates that the addition of a surfactant can significantly improve GO’s adsorption performance. It is important to note that to the best of our knowledge, no investigations have been reported in the literature regarding the use of surfactant-modified GO for PFAS removal.

Thus, for the purpose of capturing PFAS in water, this study compared the adsorption performance of seven modified GOs with the unmodified one in terms of removing a mixture of 11 PFAS in water. Detailed studies were then carried out for GO modified by a cationic quaternary ammonium surfactant, cetyltrimethylammonium chloride (CTAC). Based upon extensive characterization by scanning electron microscopy with energy dispersive X-ray spectroscopy (SEM-EDS), Fourier transform infrared spectroscopy (FTIR), thermogravimetric analysis (TGA), X-ray diffraction (XRD), Brunauer–Emmett–Teller (BET), and X-ray photoelectron spectroscopy (XPS) and in-depth studies of adsorption kinetics, isotherms, and impact from environmental factors, the mechanisms underlying PFAS adsorption by GO-CTAC were proposed. Finally, the adsorption of PFAS in river water samples by GO-CTAC was evaluated.

## 2 Materials and Methods

### 2.1 Materials

The details of the chemical reagents and the physicochemical features of PFAS used in this study are shown in Tables S1 and S2, respectively. The river water was collected from the adjacent Hudson River at Albany, New York, U.S., and stored at 4 °C before use. The composition of the river water is listed in Table S3. Milli-Q water (resistivity ≥ 18.2 MΩ·cm) was used to prepare solutions throughout the experiment.

### 2.2 Synthesis of Graphene Oxide Modified by Different Reagents

GO exhibits high susceptibility to surface modifications due to its chemical reactivity. To investigate which modification of GO is effective for removing PFAS from aqueous solutions, different types of functionalized GO-based adsorbents were synthesized following a procedure reported by Yang et al.,^30^ with minor adjustments. Details were provided in Text S1 for reduced GO and Texts S2–S6 for modified GO. In brief, to modify GO, a solution was prepared by dispersing a certain amount of graphene oxide (Sigma-Aldrich) in 50 mL of water, along with a fixed mass of a chosen reagent, such as ethanolamine (EA), diethylene triamine (DETA), hexamethylenetetramine (HMT), poly(diallyldimethylammonium chloride) (PDDA), or cetyltrimethylammonium bromide (CTAB). Different mixing schemes, dry temperatures, and drying times were then used to obtain GO-EA, GO-DETA, GO-HMT, GO-PDDA, and GO-CTAB, respectively. In terms of making GO-CTAC, 30 mg of CTAC was added to 15 mg of GO in 50 mL of deionized water. The mixture was then subjected to ultrasonic agitation for a duration of 4 h. The suspension was allowed to stay undisturbed for 24 h, during which time it naturally separated into distinct layers. After the suspension was centrifuged at 4500 rpm for 15 min, the solid was gathered and subjected to freezing at −20 °C for 24 h, after which it was freeze-dried at −45 °C for 72 h. Similarly, the resulting composite is denoted as GO-CTAC in the subsequent sections of the study. The same GO was used directly as a control for various studies without any modifications. Once synthesized, these adsorbents were tested for removal of a mixture of PFAS, each at an initial concentration of 10 μg/L. Subsamples were collected at 1, 8, 24, and 48 h.

Detailed information on adsorbent characterization, PFAS adsorption experiment, kinetics and isotherm experiments, environmental variables, river water adsorption investigations, and PFAS analysis is presented in Texts S7–S10 in the Supporting Information.

### 2.3 Adsorption Kinetics and Isotherms Modeling

In order to assess the adsorption capabilities of GO-CTAC and compare the adsorption rates of various PFAS, three commonly used kinetic models – namely, pseudo-first-order (PFO), pseudo-second order (PSO), and intraparticle diffusion (IPD) models were employed to analyze and fit the adsorption data.^31^ The equations are shown below
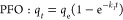
1
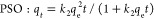
2
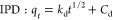
3where *t* (h) represents the contact time, *q_t_* (mg/g) stands for the amount of adsorbate in the solid phase at time *t*, and *q*_e_ (mg/g) stands for the adsorbed amount at equilibrium. The rate constants for the PFO and PSO, *k*_1_ (h^–1^) and *k*_2_ (g/(mg·h)), respectively, the IPD coefficient, *k*_d_ (mg/(g·h^1/2^)), and the constant associated with the boundary layer thickness, *C*_d_ (mg/g), are defined by the experimental data.

The adsorption data was also fitted using four different isotherm models, namely the Langmuir, Freundlich, Sips, and Toth models, as shown in eqs 4 through 7, in that order^32^
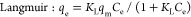
4
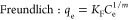
5
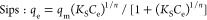
6
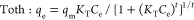
7where *q*_e_ (mg/g) represents the adsorbed amount at equilibrium: adsorbate (mg)/sorbent (g) at equilibrium, whereas *q*_m_ (mg/g) denotes the maximum adsorption capacity; *C*_e_ (μg/L) defines the concentration of the adsorbate in the aqueous phase at equilibrium. *K*_L_ (L/μg) is the Langmuir constant related to the adsorption capacity. *K*_F_ (mg·L^1/m^/(g·μg^1/m^)) is the Freundlich constant, which is associated with both adsorption capacity and energy. *K*_S_ (L/μg) is the Sips constant, representing adsorption affinity. *K*_T_ (L/μg) is the Toth isotherm constant. The adsorption process favorability is represented by the dimensionless heterogeneity parameter *m*; the heterogeneity factor is explained by *n*; and *t*, a constant, characterizes the adsorption system heterogeneity.

## 3 Results and Discussion

### 3.1 Selection of Adsorbents

**Figure 1:**
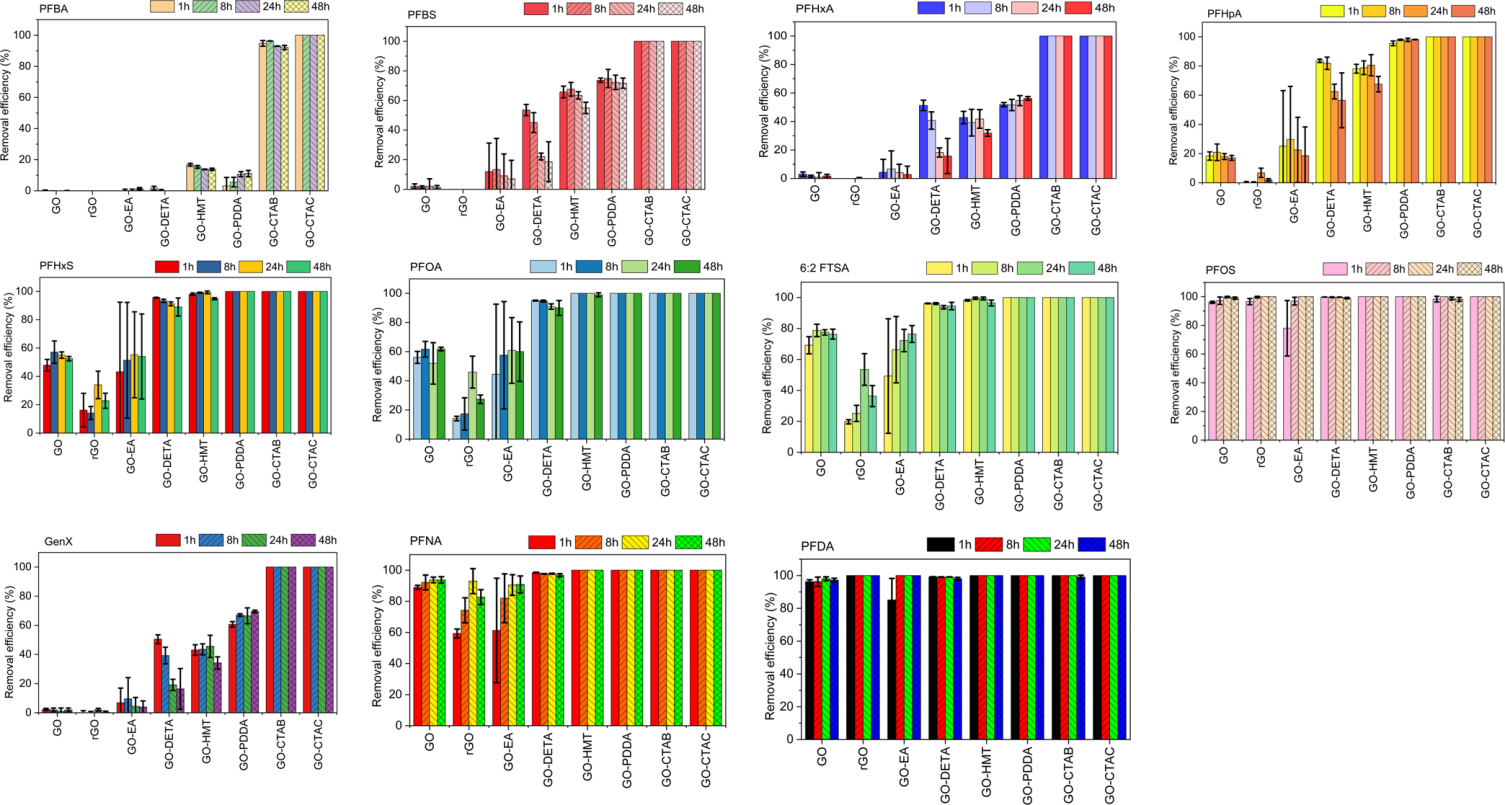
Adsorption of PFAS by GO-based adsorbents at 1, 8, 24, and 48 h. Initial PFAS concentration: 10 μg/L; adsorbent dose: 100 mg/L. Error bars indicate the standard deviations of triplicate measurements.

As shown in Figure 1, in the presence of pure GO, the removal efficiency was 50–60% for PFHxS, PFOA, and 6:2 FTSA, while it reached 90% for PFOS, PFNA, and PFDA after 48 h. Although the surface charge of pure GO is negative, significant adsorption was observed due to nonelectrostatic interactions between GO and PFAS.^26^ Compared to GO, the rGO derived from the reduction of GO by ascorbic acid had similar adsorption efficiency for PFOS and PFDA. However, its adsorption of PFHxS, PFHpA, 6:2 FTSA, PFOA, and PFNA was much poorer than pure GO’s. Therefore, the rGO was not further studied.

Adsorbents that are functionalized with amines provide alternate methods for controlling PFAS in wastewater.^33^ Therefore, we synthesized three amine-modified GO adsorbents in order to examine their effectiveness in removing PFAS. GO-EA demonstrated removal rates of about 100% for PFOS and PFDA, over 80% for PFNA, and less than 70% for PFHxS, PFOA, and 6:2 FTSA after 24 h. The functionalization of GO with DETA led to around 90% removal of PFHxS, PFOA, 6:2 FTSA, PFOS, PFNA, and PFDA, 80% removal of PFHpA, but much less removal observed for other short-chain PFAS. The overall adsorption of all PFAS was improved when the GO was chemically modified with hexamethylenetetramine (HMT). After 24 h, a removal efficiency of almost 100% was achieved for PFHxS, PFOA, 6:2 FTSA, PFOS, PFNA, and PFDA. The removal of the other PFAS was less than 80%. The enhanced adsorption of relatively hydrophobic PFAS could be due to additional active sites provided by HMT.^34^

Given the negative charges inherent to the tested PFAS at environmentally relevant concentrations, adsorbents with a cationic nature have been reported to remove PFAS rapidly due to strong electrostatic attractions.^35^ In this study, cationization demonstrated superior removal efficacy compared to amine functionalization. For instance, during 1 h, a removal efficiency of over 95% was seen for PFHxS, PFOA, 6:2 FTSA, PFOS, PFNA, and PFDA. Additionally, a removal efficiency of over 90% was achieved for PFHpA and over 80% for PFBS. Furthermore, PFBA and GenX showed a removal effectiveness of around 50%. This suggests that the quaternary ammonium functional groups of PDDA are not fully convinced of PFAS removal performance; thereby, modification of GO is still a reasonable effort in terms of PFAS removal performance.^36^

Surfactant modification plays an important role in altering the surface chemistry of the adsorbents and increasing their affinity for PFAS capture.^37,38^ Besides, surfactant-modified natural zeolites were 3-fold less expensive than activated carbon, which is promising for developing surfactant-based adsorbents.^39^ Accordingly, at first, GO was modified with CTAB (a cationic surfactant), and the resulting GO-CTAB achieved almost 100% removal of all tested PFAS within 1 h, except for PFBA, whose 95% removal efficiency after 8 h suggested room for further improvement. CTAC is structurally similar to CTAB. The GO-CTAC sorbent, however, was able to remove about 100% short- and long-chain PFAS within 1 h. CTAC may serve as a bridge to decrease the repulsion between PFAS molecules. This allows PFAS to form micelle structures at concentrations lower than its CMC (critical micelle concentration) and enhances the adsorption capacity of PFAS via the process of micelle aggregation.^40−42^ The results of all screening tests thus yielded GO-CTAC as the best adsorbent, exhibiting almost 100% removal of all target PFAS. This observation can be correlated with previous literature, which shows that the CTAC addition enhanced PFAS removal performance.^43^ As detailed below, an extensive investigation was conducted to fully comprehend the adsorption performance and processes involving GO-CTAC.

### 3.2 Characterization of Adsorbents

#### 3.2.1 Morphological and Physicochemical Properties

**Figure 2:**
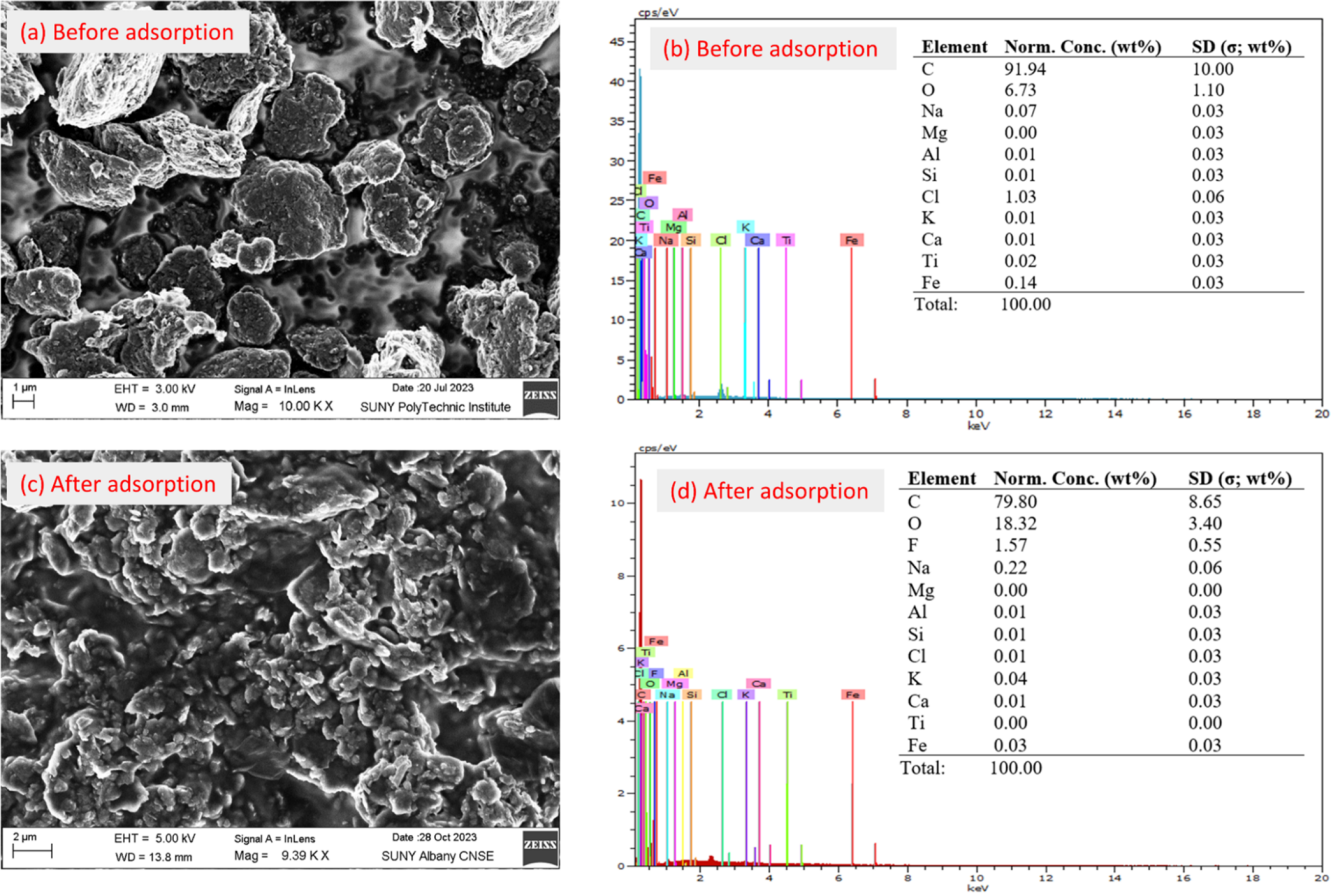
Morphological and compositional analyses of before adsorption (a, b) and after adsorption (c, d) by SEM-EDS. Elemental compositions are shown with normalized concentrations (wt %) and standard deviations (SD).

Scanning electron microscopy (SEM) was used to investigate the surface morphology of GO-CTAC before and after PFAS adsorption. Before adsorption, the GO-CTAC appeared as large, agglomerated particles with irregular shapes formed by numerous tiny particles (Figure 2a). The EDS analysis (Figure 2b) indicated that carbon (C), oxygen (O), and chlorine (Cl) constituted more than 98% of the overall elemental composition.^44^ Following the adsorption process, the adsorbent surface assembled into a more homogeneous and denser network structure, which could play a significant role in PFAS adsorption by facilitating the diffusion of the adsorbate through cavities (Figure 2c). Besides, the principal elements exhibited minor fluctuations due to the exchange of ions between PFAS compounds and water molecules coordinated with GO-CTAC.^45,46^ Moreover, the EDS spectrum identifies the presence of fluorine (F) (1.57%) originating from the adsorbed PFAS, meaning successful PFAS adsorption onto the GO-CTAC (Figure 2d).

Interestingly, it was observed that the particle size underwent a reduction after the cationic modification process with values of 2.75 ± 0.81 μm for GO and 0.69 ± 0.79 μm for GO-CTAC. Table S5 lists the BET-specific surface area, total pore volume, and maximum pore width of pristine and CTAC-modified GO. It was noticed that with the inclusion of CTAC, GO’s pores were gradually occupied by this cationic polymer. Prior research has also shown that CTAC addition to the GAC surface reduced BET-specific surface area and total pore volume and improved the bromate adsorption capacity.^47^

#### 3.2.2 Crystallinity

**Figure 3:**
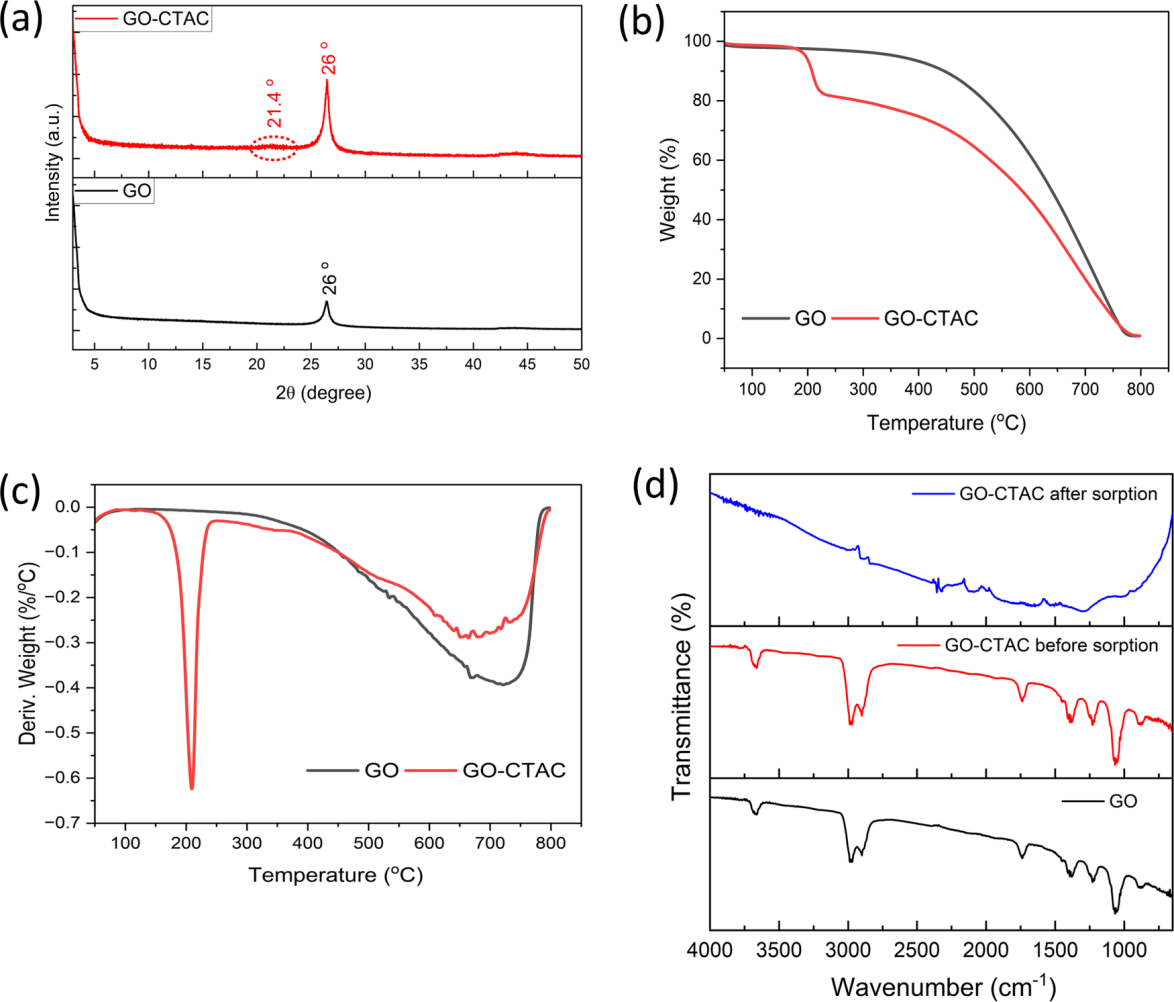
(a) XRD, (b) TGA, (c) DTG, and (d) FTIR spectra of the GO and GO-CTAC (before adsorption) and GO-CTAC (after adsorption) of GO and GO-CTAC, respectively.

The X-ray diffraction (XRD) analyses of both GO and GO-CTAC are shown in Figure 3a. The XRD pattern of GO reveals a small peak at 2θ = 26°, indicating the presence of (002) sheets in this particular form of graphene oxide with an interlayer spacing of around 3.36 Å. The presence of the same distinct peak at 2θ = 26° in the CTAC-loaded GO suggests that the addition of CTAC to the GO did not impact its crystallinity. Nevertheless, the peak exhibited greater sharpness when the interlayer distance was increased compared to the pure GO. In addition, a distinct and wide peak at around 21.4° was detected, indicating the presence of CTAC.^48^ This observation provides evidence that CTAC was effectively loaded onto the GO material.

#### 3.2.3 Thermal Behaviors

Figure 3b,3c represent the TGA curves and their first derivatives (DTG) to investigate the thermal stability of GO and GO-CTAC, respectively. As anticipated, GO demonstrated thermal stability and had a slight 2.4% reduction in weight up to 800 °C in the presence of N_2_ gas. This phenomenon might be attributed to the evaporation of the remaining adsorbed and bound water that filled the gaps between the graphene oxide layers.^49,50^ For GO-CTAC, a mere 2.2% reduction in weight was observed below 120 °C due to the removal of residual water, suggesting that CTAC has effectively replaced water in the gaps between the layers of GO.^51^ The first significant reduction in mass of GO-CTAC at around 210 °C mostly results from the breakdown of the oxygen-containing functional groups.^52^ Additionally, the pyrolysis of CTAC was observed, resulting in a 20% decrease in weight until reaching 300 °C. While the weight reduction of GO-CTAC was more than that of GO, the residual char of the GO-CTAC sample was higher than that of GO, indicating that CTAC plays a significant role in stabilizing the GO composite.

#### 3.2.4 FTIR

The functional group characteristics of GO, GO-CTAC, and PFAS-laden GO-CTAC were investigated by using FTIR analysis, as shown in Figure 3d. In the FTIR spectra of GO, a band between 3750 and 3700 cm^–1^^53^ was observed, which corresponds to the stretching and bending vibration of OH groups of H_2_O molecules adsorbed on GO. The asymmetric and symmetric stretching vibrations of CH_2_ bonds are represented by sharp bands at 2990 and 2850 cm^–1,^^54^ respectively. The stretching vibrations of carbonyl (C=O) and aromatic C=C groups^55^ of GO are attributed to the characteristic bands around 1750 and 1573 cm^–1^, respectively. Additionally, the band close to 1370 cm^–1^ indicates the presence of hydroxide groups (C–OH)^56^ on the GO. The stretching vibration of C–O–C of the alkoxy group can be observed at a strong FTIR frequency of 1030 cm^–1^, while the deformation of the aromatic C–H group can be observed at 890 cm^–1^.^57,58^ GO-CTAC displays all of the characteristic bands of GO, with strong signals that may be attributed to CTAC’s slight structural impact. No particular FTIR band of CTAC was detected in the GO-CTAC. However, Mehta et al. have reported that the typical FTIR band for CTAC is around 1243 cm^–1^, corresponding to the C–N group’s stretching vibrations. This band could serve as an indicator of quaternary ammonium (N^+^) salt.^59^ After PFAS adsorption, the intensity of a few FTIR bands of GO-CTAC was reduced, and new characteristic bands appeared, as shown in the FTIR spectra of PFAS-laden GO-CTAC. The interactions of organic fluorine and sulfonate of PFAS with GO-CTAC reduced the FTIR bands at 1500–1000 cm^–1^ to one band.^60^ In addition, the hydrophobic −CH_2_– FTIR bands of GO were decreased. Overall, the FTIR bands indicated that strong PFAS adsorption on the surface of GO-CTAC was due to both electrostatic and hydrophobic interactions.

#### 3.2.5 XPS

Figure S1 displays the X-ray photoelectron spectroscopy (XPS) survey spectra of the samples taken before and after adsorption, which allowed us to delve further into the process of binding of PFAS to GO-CTAC. The primary constituents detected on the GO-CTAC surface (Table S6) were carbon (87.58%), oxygen (10.58%), and nitrogen (1.45%). In addition, a chlorine (Cl) 2p signal with a relative abundance of 0.39% was observed in the GO-CTAC sample. The findings demonstrate the effective grafting of the quaternary ammonium group onto the surface of GO. A prominent F 1s (11%) peak in the XPS scan upon adsorption indicates that GO-CTAC has a robust ability to adsorb PFAS, suggesting a high adsorption tendency.

**Figure 4:**
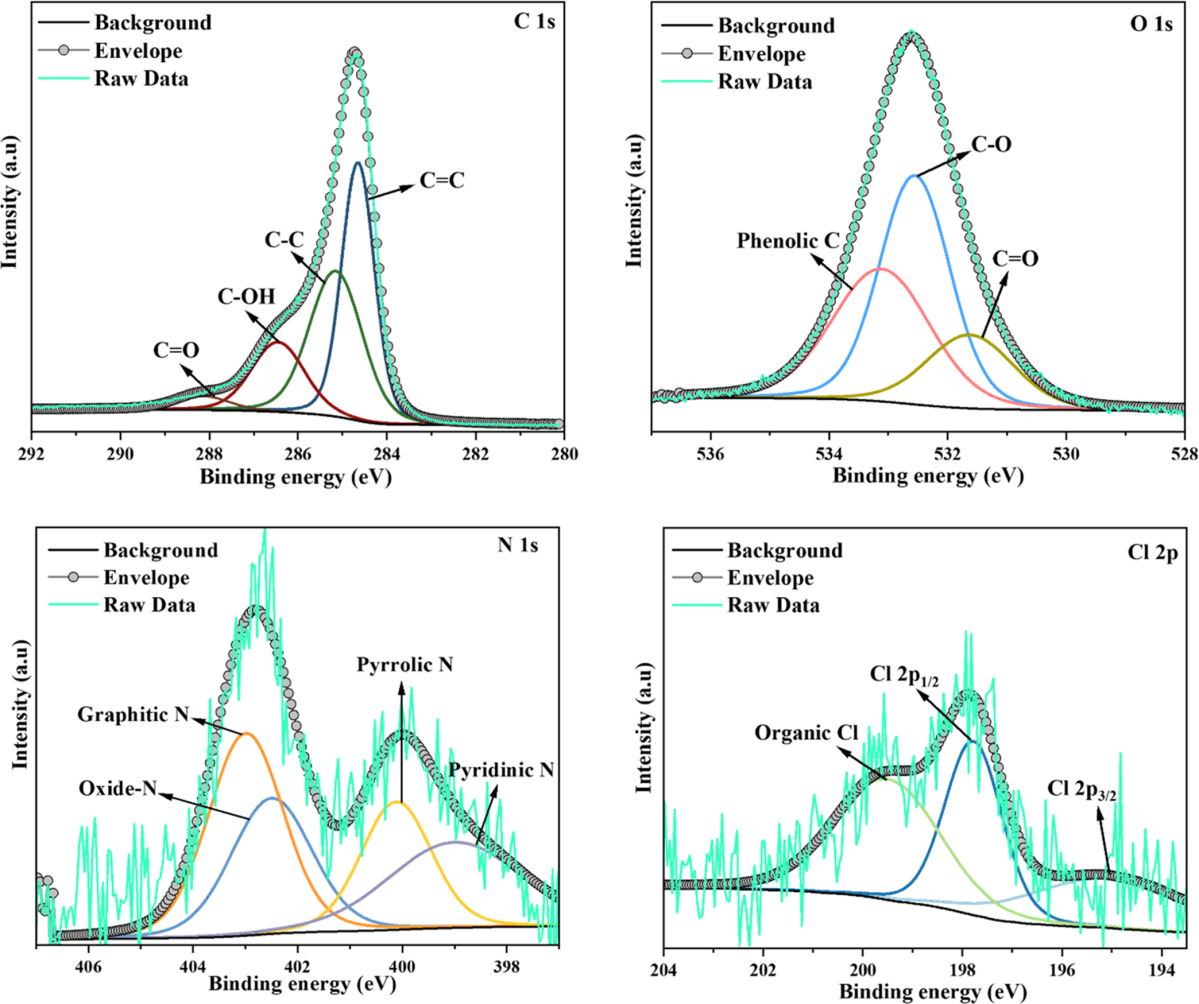
C 1s, O 1s, N 1s, and Cl 2p spectra of GO-CTAC (before adsorption).

**Figure 5:**
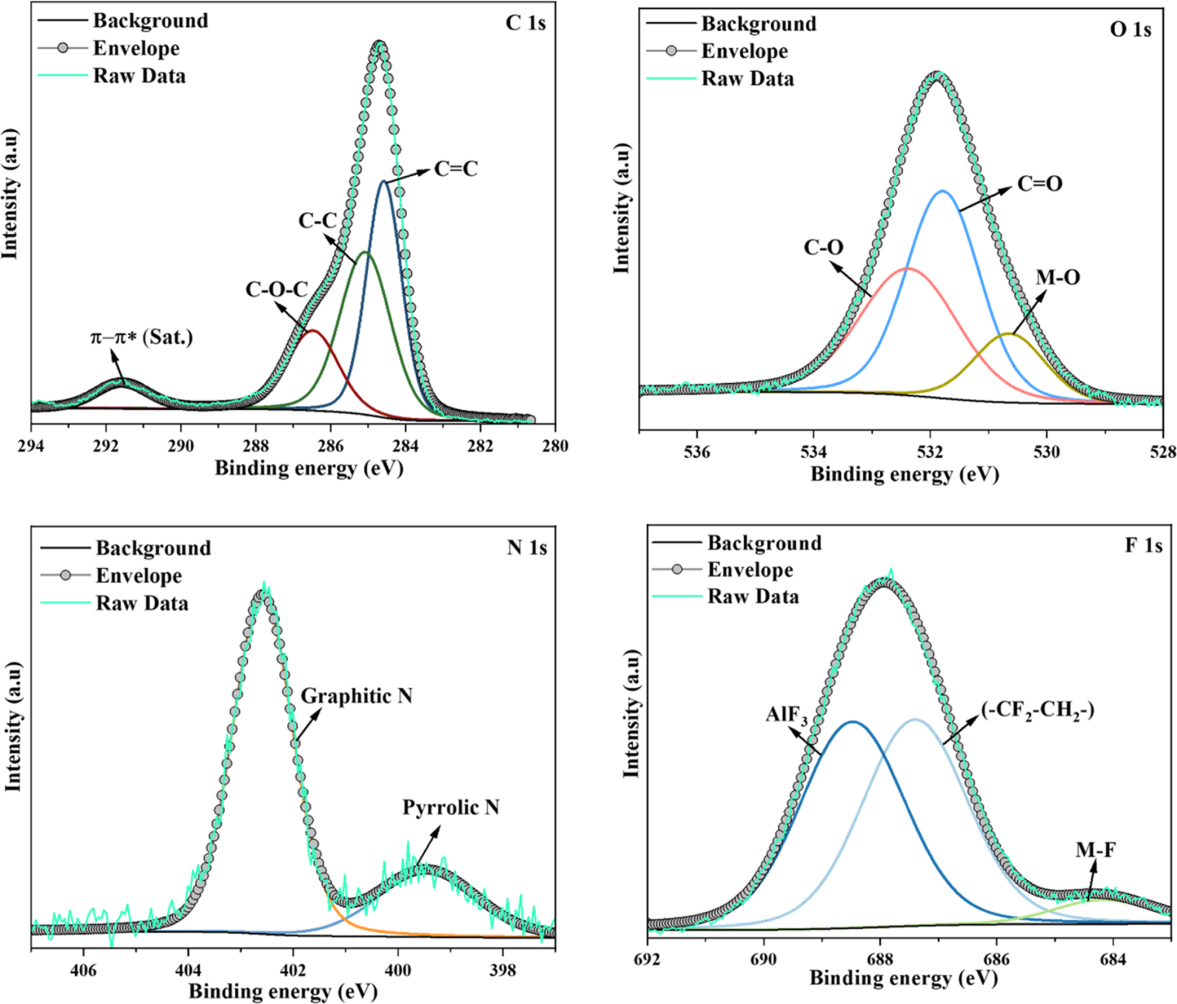
C 1s, O 1s, N 1s, and F 1s spectra of GO-CTAC (after adsorption).

**Figure 6:**
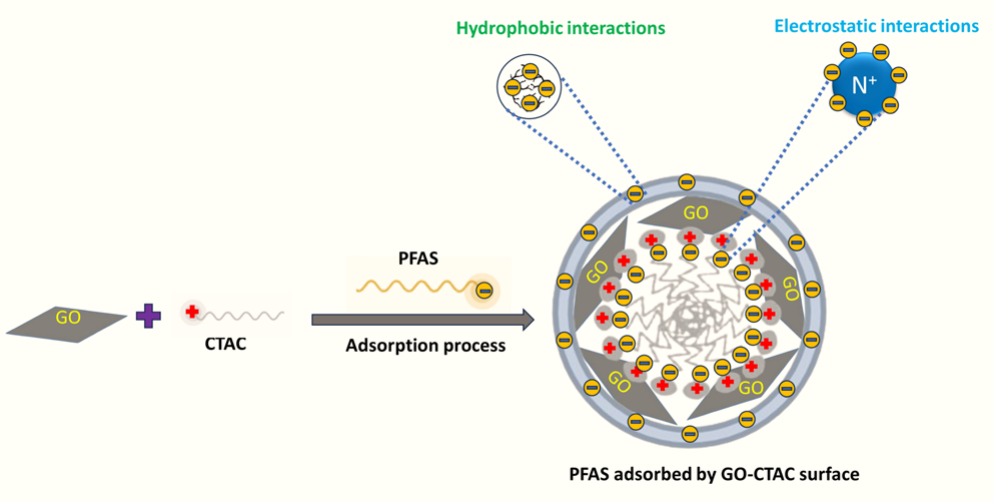
Plausible mechanism of PFAS adsorption by GO-CTAC.

Figure 4 shows the high-resolution spectra of C 1s, O 1s, N 1s, Cl 2p, and F 1s before and after PFAS adsorption, respectively. The C 1s spectra of GO-CTAC (before adsorption) exhibit peaks at 284.6, 285.3, 286.2, and 288.2 eV, which may be attributed to the presence of C=C, C–C, C–OH, and C=O bonds, respectively.^61^ Following adsorption (Figure 5), the peak at 286.2 eV was identified as C–O–C, a characteristic often seen in graphite oxide structures,^62^ resembling C–OH. A weak peak at 291.5 eV is attributed to the π–π* shakeup transition, demonstrating that CTAC assembles on GO surfaces via π–π interactions.^63^ Similarly, the spectra of O 1s may be readily differentiated before and after adsorption, as seen in Figures 4 and 5. The primary binding energies of 531.8 and 532.7 eV are attributed to the C=O and C–O groups at the edges.^64^ The signal at 400.2 eV in the N 1s spectrum indicates the presence of C–N bonding, which may generate lone pairs in the sample.^65^ The emergence of a distinct peak at 402.3 eV in the N 1s spectrum of GO-CTAC may be attained due to the presence of the quaternary ammonium groups in CTAC (Figure 4). The C–N peak exhibited a change from 400.2 to 399.4 eV upon saturation of PFAS on GO-CTAC (Figure 5). The alteration of the C–N bond characteristics is likely a result of the adsorption of PFAS by the amine groups. Figure 4 displays the XPS spectrum of Cl 2p. The GO-CTAC samples exhibited Cl 2p1/2 and Cl 2p3/2 peaks at 197.7 and 195.6 eV, respectively. The difference in binding energy between these peaks was measured to be 2.1 eV due to the presence of chloride in the quaternary ammonium groups.^66^ The atomic percentage of chlorine decreased from 0.39 to 0.07% after the adsorption of PFAS. Furthermore, the F 1s spectra (Figure 5) exhibited a prominent peak at 688.7 eV, indicating that ion exchange played a significant role in the adsorption of PFAS. Nevertheless, since the removal efficiency of PFAS was not strongly affected by the presence of the NaCl solution (Figure 9b), it can be inferred that additional adsorption mechanisms, such as hydrophobic interactions and electrostatic attraction, played a crucial role in the adsorption process (Figure 6).

### 3.3 Adsorption Performance

#### 3.3.1 Adsorption Kinetics

**Figure 7:**
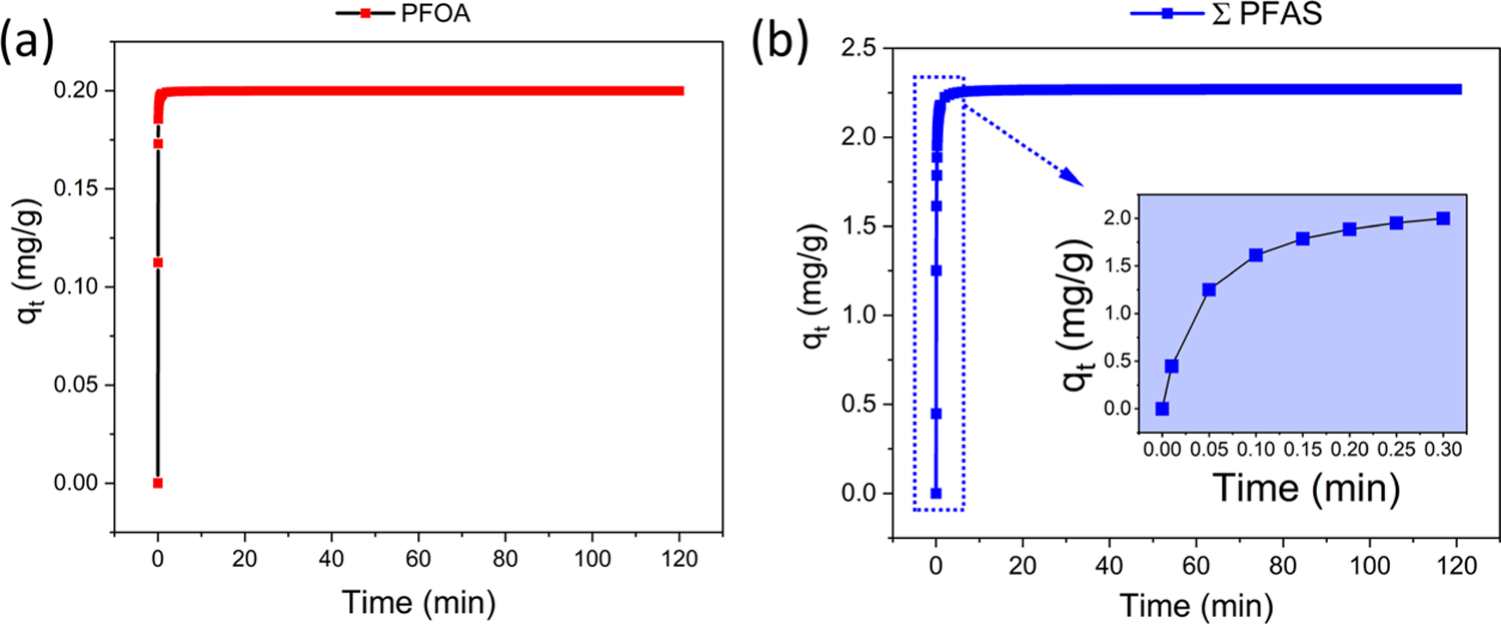
(a) Representative kinetic curve of PFOA adsorption and (b) total PFAS adsorption kinetic curve at initial concentrations of 20 μg/L by GO-CTAC. The colored circles represent experimental data at each time point, and the colored solid lines indicate fitting data by the PSO model. Experimental data represent the mean values of triplicate measurements.

Adsorption kinetics was examined to determine the adsorption rate of GO-CTAC for PFAS removal at an initial concentration of 20 μg/L. As indicated by Figure 7a, for PFOA (as a representative for all PFAS) and total PFAS, significant adsorption occurred in the first 30 s (Figure 7b, inset), and the adsorption equilibria were reached within 5 min. In comparison to the reported equilibration time for granular activated carbon (4–240 h), an ion-exchange resin (2–168 h), and the majority of powdered carbon materials (1–24 h), the adsorption of PFAS by GO-CTAC was rapid^67^ (Table S7). Figures S2 and S3 show that the squared correlation coefficients *R*^2^ for the 11 PFAS were 0.0001 and 0.175–0.322 for the linearized PFO and IPD models, respectively. Conversely, the linearized PSO model exhibited strong fitting correlations, with *R*^2^ values ranging from 0.900 to 1 for the 11 PFAS (Figure S4). This suggests that the PSO models were able to describe the adsorption kinetics of all of the studied PFAS well. Similar kinetics trends were observed by Chen et al.,^68^ who demonstrated that the PSO model provides the optimum adsorption rate for PFOA and PFOS by carbonate-layered double hydroxides. These findings indicate that the chemisorption mechanism was the main factor in the adsorption process, and GO-CTAC and PFAS molecules might have shared or exchanged electrons at their binding sites.^69^

#### 3.3.2 Adsorption Isotherm

**Figure 8:**
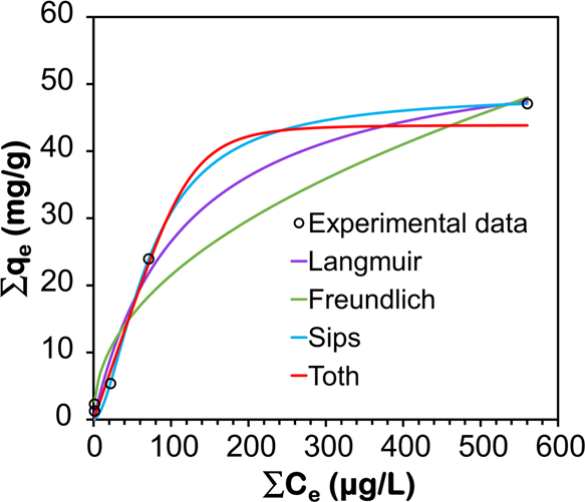
Adsorption isotherms of total PFAS by GO-CTAC, fitted by the Langmuir, Freundlich, Sips, and Toth models. Experimental data represent the mean values of triplicate measurements.

Several isotherms have been used to reveal the maximum adsorption capacity of GO-CTAC. The Langmuir isotherm explains the adsorption and desorption in a dynamic system, while the Freundlich isotherm characterizes the adsorption process on heterogeneous surfaces. The Sips and Toth models integrate both isotherms, predicting adsorption behaviors in heterogeneous systems.^70^ The *q*_e_ and *C*_e_ values in Figure 8 were computed for the total concentration of PFAS (∑PFAS) and were utilized to develop isotherm models. The correlation between the initial concentration (*C*_0_) and the amount of PFAS absorbed by the GO-CTAC is shown in Figure S5. Figure 8, Tables S8, and S9 demonstrate that the Langmuir model accurately represented the experimental adsorption data at high concentrations, but the Freundlich isotherm offered an exceptional match at low concentrations. The Sips and Toth isotherms effectively and accurately represented all of the data. The Sips isotherm tackles the constraints related to a rising adsorbate concentration in the Freundlich model. At low adsorbate concentrations, it exhibits behavior comparable to that of the Freundlich model and predicts the adsorption of a single layer, resembling the Langmuir model at high adsorbate concentrations. Comparing the Sips and Toth isotherms, the Sips isotherm exhibited a better fit with an *R*^2^ value of 0.998, greater than 0.993 of the Toth’s. Previous studies also showed that the Sips model is more suitable for explaining PFAS adsorption behavior.^71,72^ According to the Sips isotherm, the highest adsorption capacity is 48.47 mg/g. In contrast to most previous research that focused on targeting just one or a few individual PFAS compounds at the milligram per liter level, the GO-CTAC used in this work showed a remarkable ability to adsorb a combination of PFAS compounds at the low μg/L levels that are ecologically relevant (Table S7). Furthermore, it was observed that within the 48 h testing period, no desorption of both short- and long-chain PFAS took place, hinting that the GO-CTAC has considerable potential for removing mixed PFAS in different environmental matrices.

#### 3.3.3 Effect of Environmental Factors

**Figure 9:**
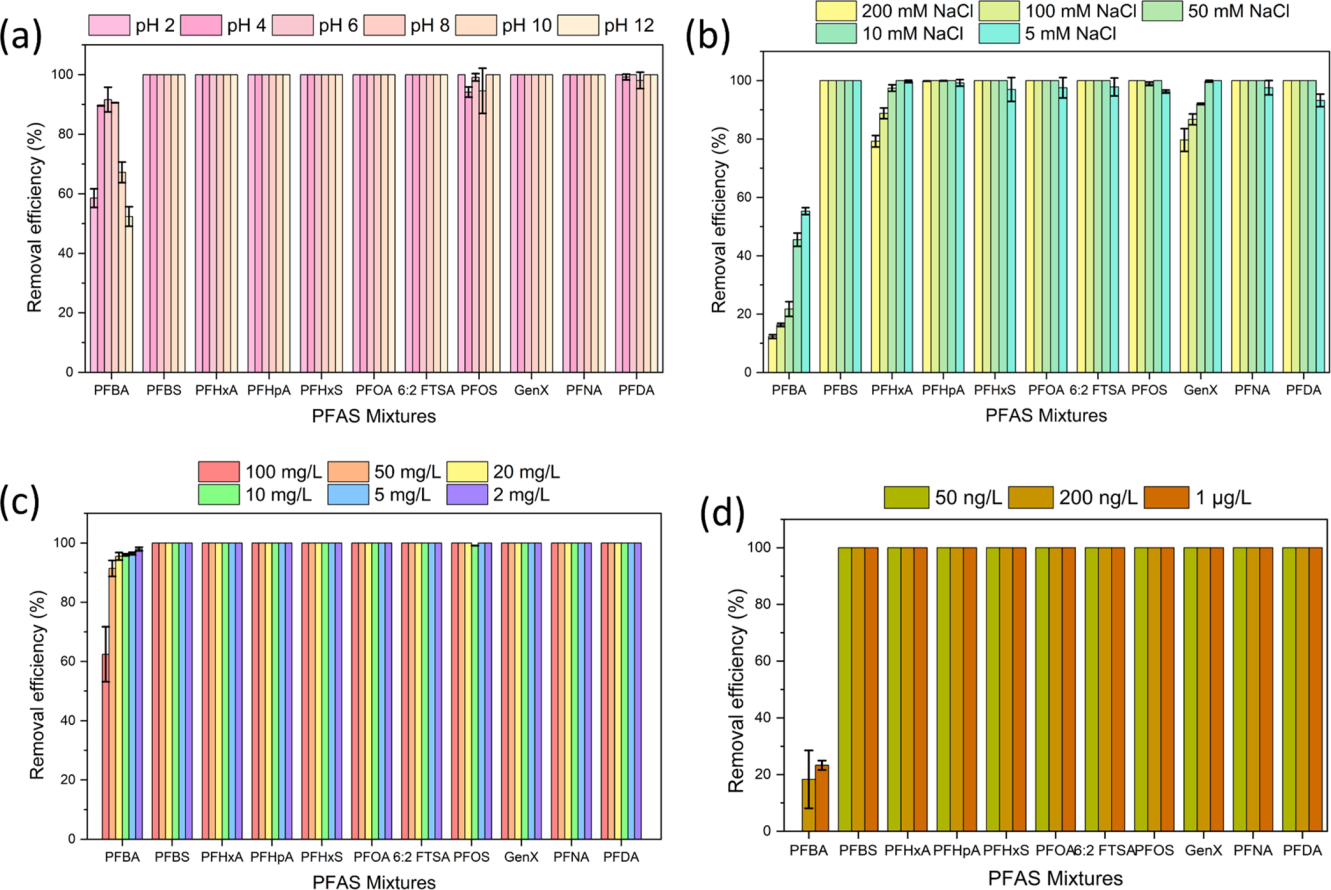
Effect of (a) pH, (b) NaCl, (c) NOM, and (d) Hudson River PFAS adsorption by GO-CTAC.

The solution pH plays an important role in determining the adsorption performance.^73^Figure 9a demonstrates that GO-CTAC had exceptional efficacy in removing all tested PFAS except PFBA at all pH ranges (2–12). This can be correlated with the point of zero charges of the adsorbent, which was consistently positive over the studied pH range (Figure S6). This observation hinted that hydrophobic interactions between GO-CTAC and PFAS were critical for PFAS removal.^74^ On the other hand, it is not surprising to see decreased PFBA adsorption by GO-CTAC at pH 10 and 12, given the rise in pH leading to loss of protons in functional groups and electrostatic repulsion between the surface of the adsorbent and anionic PFAS compounds. The lower adsorption of PFBA at pH 2 compared to those at pH 4–8 could also be due to electrostatic repulsion, as both GO-CTAC and PFBA were strongly protonated. This is in line with decreased PFBA adsorption at pH 3 compared to pH 5–9, as reported by Min et al.^11^ Overall, GO-CTAC could be a viable adsorbent for removing PFAS in a wide range of pH from water media.^75^

The impact of the ionic strength on the adsorption of PFAS by GO-CTAC was examined by varying the amounts of NaCl (5–200 mM) (Figure 9b). For short-chain PFAS, namely PFBA, PFHxA, and GenX, the correlation between adsorption and ionic strength between 50 and 200 mM was negative; the higher the ionic strength, the lower the adsorption. Prior research has shown that cations can attach to anionic functional groups of PFAS, forming weak neutral complexes. This process decreases PFAS adsorption by lowering possible electrostatic interactions with the adsorbent.^76,77^ Remarkably, for the long-chain PFAS, % removal was observed in the range of 5–200 mM. This confirms that the GO-CTAC could be a suitable adsorbent for capturing PFAS in surface water, groundwater, and seawater with an ionic strength of 1–5, 1–20, and 700 mM, respectively.^78^

As shown in Figure 9c, GO-CTAC consistently demonstrated high adsorption with humic acid ranging from 2 to 100 mg/L for all target PFAS except PFBA with humic acid at 100 mg/L. The negative impact of humic acid on PFBA adsorption suggested competition between humic acid and these four-carbon PFAS for adsorption sites on the GO-CTAC surface. For instance, the presence of organic matter greatly reduced the adsorption of PFAS by boehmite.^79^ Furthermore, the NOM, with its negative surface charges, could hinder the movement of negatively charged PFAS anions by means of electrostatic repulsion. Nevertheless, it is noteworthy that even at a higher concentration of 100 mg/L, GO-CTAC effectively eliminated almost 100% of PFAS from the water. This discovery suggested that the GO-CTAC showed potential for successful PFAS cleanup in surface and groundwater where NOM is typically around 2–10 mg/L.^80,81^

To accurately evaluate the usefulness of GO-CTAC toward PFAS removal in real-world applications, adsorption studies were carried out using water collected from the Hudson River. The river water contained a few PFAS in the range of 5–25 ng/L (Table S3). As shown in Figure 9d, almost 100% removal was observed for all spiked PFAS at 50, 200, or 1 μg/L in 4 h except PFBA, for which the removal decreased from 23 to 18 to 0% when the starting concentration of PFBA varied from 1 μg/L to 200 and 50 ng/L, respectively. The GO-CTAC thus exhibited a notably reduced adsorption capacity for PFBA in river water compared to that in pure water (Figure 1). This suggests that the presence of chemicals in the river water adversely impacted the efficacy of the GO-CTAC toward PFBA. As indicated in Table S3, the collected river water had bromide at 5.64 × 10^–3^ mg/L and phosphate at 7.60 × 10^–3^ mg/L. Its TN content of 1 mg/L was higher than that in precipitation and stormwater.^82,83^ The TOC concentration was comparable to those in surface water worldwide, which is around 5.578 mg/L.^84^ Anions and TOC are recognized to compete for the adsorption sites with PFAS and could detrimentally affect the adsorbent’s performance.^69,85^ Unlike other studies that have shown a decreased removal of PFAS from natural water by other adsorbents,^86,87^ the GO-CTAC has great potential for capturing PFAS in realistic water environments.

## 4 Conclusions

This work systematically prepared a series of GO-based materials, and GO-CTAC was found to be the best adsorbent for the removal of PFAS from water media. Characterization results demonstrated that CTAC had been successfully loaded onto the GO surface without changes in their properties. Notably, the addition of CTAC led to a highly positive surface charge of the GO-CTAC adsorbent, thus ultimately playing a critical role in the adsorption efficiency. The results showed that GO-CTAC was able to capture almost 100% of target PFAS in pure water in 1 h. Its effectiveness for PFAS removal, except PFBA, was not affected much by changes in pH, concentration of NOM, and ionic strength. When added to river water samples, the removal of ten PFAS was almost 100% in 4 h. GO-CTAC’s adsorption of PFAS could be described accurately by a Sips isotherm model with a computed adsorption capacity of 48.47 mg/g. Based on the physicochemical properties of the GO-CTAC and results from the study of environmental impact on PFAS adsorption, adsorption mechanisms involving electrostatic and hydrophobic interactions were proposed. This study thus adds an interesting, fast-acting, and well-tolerating adsorbent to the current search for effective materials for removing PFAS in various water environments.

## Supporting Information Available

Chemical and reagent information; PFAS physiochemical characteristics, experimental adsorption; isotherm, and kinetics details; PFAS analysis, adsorption data, isotherm, and kinetics analysis (PDF)
