# Supplementary material for: Surface Modification of Graphene Oxide for Fast Removal of Per- and Polyfluoroalkyl Substances (PFAS) Mixtures from River Water
Source: ACS ES T Water. 2024 Jun 11;4(7):2968–80. doi: 10.1021/acsestwater.4c00187 (PMC11249979; doi:10.1021/acsestwater.4c00187)
Supplement: Supplementary file 1 — ew4c00187_si_001.pdf [file ew4c00187_si_001.pdf]

Supporting Information for

Surface Modification of Graphene Oxide for Fast Removal of Per- and Polyfluoroalkyl Substances (PFAS) Mixtures from River Water

Md. Nahid Pervez [a], Tao Jiang [a,*], Jaydev Kumar Mahato [a], Aswin Kumar Ilango [a], Yamini Kumaran [b], Yuwei Zuo [a], Weilan Zhang [a], Haralabos Efstathiadis [b], Jeremy I. Feldblyum [c], Mehmet V. Yigit [c], Yanna Liang [a]

[a] *Department of Environmental and Sustainable Engineering, University at Albany, State University of New York, Albany, New York 12222, United States*

[b] *Department of Nanoscale Science and Engineering, University at Albany, State University of New York, Albany, New York 12222, United States*

[c] *Department of Chemistry, University at Albany, State University of New York, Albany, New York 12222, United States*

* Corresponding author:

tjiang2@albany.edu (Tao Jiang)

Text S1. Preparation of reduced graphene oxide (rGO)

The synthesis of rGO was conducted following the technique described by De Silva et al. (De Silva et al., 2018) with some minor adjustments. In summary, a solution of GO at a concentration of 0.1 mg/mL was prepared by subjecting dried GO to sonication in distilled water. The 100 mg of ascorbic acid was added to a 100 mL solution. The pH of the medium was modified to about 10 by introducing an NH_4OH solution in order to enhance colloidal stability via electrostatic repulsion (Fernández-Merino et al., 2010). The mixture was stirred at 65 °C. The resultant suspensions were subjected to centrifugation at a speed of 4500 revolutions per minute for a duration of 15 min. The solid was gathered and subjected to freezing at a temperature of -20 °C for 24 h, after which it underwent freeze-drying at -45 °C for 72 h. The resulting composite is denoted as rGO in the subsequent sections of the study.

Text S2. Preparation of ethanolamine (EA) functionalized graphene oxide (GO)

A GO-EA composite was synthesized using the approach described by Ma et al. (Ma et al., 2018) with some minor adjustments. In summary, a 50 mg sample of GO was evenly distributed in 10 mL of water using ultrasonication for 7 h. Subsequently, the solution was put into a 50 mL autoclave walled with Teflon. Then, 1 g of EA dissolved in 19 mL of water was promptly introduced into the suspension. Ultimately, the solution mixture was sealed and subjected to a temperature of 80 °C for a duration of 10 h. After undergoing natural cooling to room temperature, the precipitate was collected and subjected to three thorough washes using ultrapure water and ethanol. It was then frozen at -20 °C for 24 h and then freeze-dried at -45 °C for 72 h. The resultant composite is denoted as GO-EA in the subsequent sections of the study.

Text S3. Preparation of diethylene triamine (DETA) functionalized graphene oxide (GO)

The GO-DETA composite was synthesized following the technique described by Jiang et al. (Jiang, L. et al., 2023), with some minor adjustments. In summary, a volume of 25 mL of a GO suspension with a concentration of 4 mg/mL was combined with a specific quantity of DETA (8 μL/mL) and agitated. Subsequently, the mixture was transferred to a Teflon reactor capable of withstanding high pressure and subjected to a reaction at a temperature of 90 °C for a duration of 16 h, resulting in the production of GO-DETA powder. Subsequently, the GO-DETA was submerged in a 25% (V/V) aqueous ethanol solution for the purpose of eliminating impurities. Ultimately, the GO-DETA was subjected to a freezing process at a temperature of -20 °C for 24 h and then underwent freeze-drying at a temperature of -45 °C for 72 h. The resultant composite is denoted as GO-DETA in the subsequent sections of the study.

Text S4. Preparation of hexamethylenetetramine (HMT) functionalized GO

The GO-HMT composite was synthesized following the technique described by Lee et al. (Lee et al., 2012), with some minor adjustments. In summary, 4000 mg of HMT was dissolved in 120 mL of the graphene oxide dispersion, which included about 100 mg of GO. The mixture was stirred magnetically at room temperature for a duration of 10 min. The resultant solution was transported to an autoclave lined with Teflon, which had a capacity of 200 mL. The autoclave was subjected to a constant temperature of 180 °C in an electric oven for a duration of 12 h. The autoclave underwent natural cooling following the reaction until it reached the ambient temperature. The black solid obtained was filtered, thoroughly washed with water, and then frozen at a temperature of -20 °C for 24 h. Subsequently, freeze-drying was carried out at a temperature of -45 °C for 72 h. The resultant composite is denoted as GO-HMT in the subsequent sections of the study.

Text S5. Preparation of Poly(diallyldimethylammonium chloride) (PDDA) functionalized GO

The GO-PDDA composite was synthesized using the approach described by Chen et al. (Chen et al., 2020), with some minor adjustments. In summary, the 1 mg/mL GO dispersion was synthesized as described before. Subsequently, a 5 mL aliquot of PDDA was introduced into the GO dispersion at ambient temperature, and the mixture was agitated for a duration of 4 h. The resultant black solid was obtained using centrifugation, rinsed with water 2-3 times, and then frozen at a temperature of -20 °C for 24 h. Subsequently, it was freeze-dried at a temperature of -45 °C for a duration of 72 h. The resultant composite is denoted as GO-PDDA in the subsequent sections of the study.

Text S6. Preparation of cetyltrimethylammonium bromide (CTAB) functionalized GO

The GO-CTAB composite was synthesized using the approach described by Yang et al. (Yang and Li, 2014) with minor adjustments. In summary, a solution was prepared by dispersing 15 mg of graphene oxide in 50 mL of water, along with 30 mg of CTAB, and subjecting it to ultrasonic agitation for a duration of 4 h. The suspension was kept undisturbed for a duration of 24 h, during which it naturally separated into distinct layers. Subject the acquired CTAC-GO suspension to centrifugation at a speed of 4500 revolutions per minute for a duration of 15 min. The solid was collected and subjected to freezing at a temperature of -20 °C for 24 h, after which it was subjected to freeze-drying at a temperature of -45 °C for 72 h. The resultant composite is denoted as GO-CTAC in the subsequent sections of the study.

Text S7. Procedure for performing PFAS adsorption experiment

The adsorption experiments were carried out using 50-mL polypropylene centrifuge tubes (Corning Inc., Corning, NY, USA) in a batch mode. The experiments were repeated three times. A PFAS combination including nine PFAAs (C4, C6-C10 PFCAs, and C4, C6, C8 PFSAs), a PFOA alternative, namely GenX, and PFOS alternative, namely 6:2 FTSA, was added to each tube. The initial concentration for each PFAS was 10 µg/L, and the dosage of each adsorbent was 100 mg/L. Before introducing an adsorbent, 500 µL of the PFAS solution was extracted from each tube. These samples were used for measurements at a time of 0 min. After the adsorbent was introduced, all tubes were placed on a rotator shaker and set to rotate at a speed of 150 rpm. Specimens were obtained from each tube at 1, 2, 4, 8, 24, and 48 h. After centrifugation, the supernatant was filtered using 0.2 µm nylon syringe filters, and PFAS were evaluated using an Agilent Technologies 1290 Infinity II LC system combined with a 6470 Triple Quad Mass Spectrometer (LC-MS/MS, Santa Clara, CA, USA).

Additionally, the adsorption of PFAS in river water was examined using a similar technique, except that each PFAS was spiked at 50 ng/L, 200 ng/L, and 1 µg/L. In addition, we assessed the effects of three environmental variables on the adsorption of PFAS. These variables include pH levels ranging from 2 to 12, varying concentrations of natural organic matter (NOM) with humic acid levels ranging from 2 to 100 mg/L, and different ionic strengths with NaCl concentrations ranging from 5 to 200 mM. The initial concentration of PFAS for each type and solution pH were 10 µg/L and 6, respectively. The duration of adsorption was 4 h. The efficacy of the adsorbents in removing PFAS by adsorption was determined by using equation (S1) in the following manner:

$$\text{Removal efficiency (\%)} = \frac{C_i - C_t}{C_i} \times 100 \qquad \text{(S1)}$$

where C_i and C_t are the PFAS concentration at initial and time (t), respectively.

Text S8. Procedures for characterizing the adsorbents

Zeiss Leo 1550 scanning electron microscope (SEM) equipped with energy dispersive X-ray spectroscopy (EDS) was used to examine the morphology, microstructure, and elemental composition of the absorbents. Functional group analysis in the adsorbent samples before and after PFAS adsorption was carried out using Fourier transform infrared spectroscopy (FTIR; PerkinElmer Spectrum 100, Waltham, MA, USA). Before analysis, the crystal material was cleaned with ethanol and DI water. A certain amount of the sample was placed on the top-plate crystal material of PerkinElmer's Universal ATR (UATR) Accessory. The spectral data were acquired within the 4,000-650 cm^{-1} spectral band with a resolution of 1 cm^{-1}. Thermogravimetric analysis (TGA) was conducted using a TGA-5500 instrument (New Castle, DE, USA) in order to assess their thermal behavior. Nitrogen gas adsorption isotherms were obtained at a temperature of 77 K using a 3Flex gas adsorption analyzer (Micromeritics, Norcross, GA, USA). Before the N_2 gas adsorption examination, the adsorbents underwent activation under decreased pressure and at a temperature of 50 °C (about 0.1 mbar) for 24 h. The Brunauer-Emmett-Teller (BET) technique was used to estimate the specific surface areas, while the pore size distributions were derived using density functional theory. The X-ray diffraction (XRD) was fitted with a graphite monochromator and a D/teX Ultra one-dimensional silicon strip detector. The investigated crystalline samples were pulverized and positioned in zero-background holders, which were subjected to scanning with a 0.01° increment. The Malvern Zetasizer Nano-ZS analyzer (Malvern Panalytical Ltd, Malvern, UK) was used to measure the particle size distribution and zeta potential at a neutral pH and room temperature. PHI Quantera II system was used to perform X-ray Photoelectron Spectroscopy to study the chemical state of the elements present and the bonding configuration at the surface of the absorbent. To include and quantify C along with other elements, a PVA binder was used to

drop coat on a Si substrate using the following procedure: 20 mg of sample was added to 100 μL of DI water and ethanol each and sonicated for 15 min. Then, 100 μL of 5% PVA solution was added and sonicated for 15 min. The solution was drop cast on Si substrate and dried for 12 h at 60 °C. All the samples were imaged and analyzed at a region where the Si substrate peak was obscure.

Text S9. Chemical analysis

The concentration of PFAS in the river water was measured using EPA Method 537.1. Concisely, a surrogate of 30 µL (30 ng, 1 mg/L) ^{13}C-perfluorohexanoic acid (PFHxA) was added to each 400 mL sample. Subsequently, the sample with spikes was introduced into a Hypersep C18 cartridge that had been prepared with methanol and deionized water. Before measurement, the PFAS retained on the C18 cartridges was eluted, and the samples were then enriched with ^{13}C-PFOS and ^{13}C-PFOA as internal standards. The Agilent LC-MS/MS was used to quantify the target PFAS in the prepared samples. The supernatant samples obtained from the adsorption experiments were analyzed similarly, except that the solid phase extraction (SPE) step was omitted. Information on PFAS measurements using the LC-MS/MS method may be found in the previous papers (Jiang, T. et al., 2023; Jiang et al., 2022; Zhang et al., 2022a; Zhang and Liang, 2022; Zhang et al., 2022b) as well as in Text S10 and Tables S2 and S3. An analysis was conducted on the anions present in the river water using a 930 Compact IC Flex equipment (Metrohm, Herisau, Switzerland) that was equipped with a conductivity detector. Anions were separated with a Metrosep SUPP 5 column manufactured by Metrohm. The elution technique included using a 1:1 mixture of 1.8 mM Na_2CO_3 and 1.7 mM $NaHCO_3$ as the eluent, with a 0.7 mL/min flow rate. Later, a solution of sulfuric acid (H_2SO_4) with a concentration of 0.05 M was used as a regeneration agent to reduce the conductivity. The 1-500 µg/L range calibration curves were constructed using a standard mixture (Thermo Fisher Scientific Inc., USA) including six anions: Cl^-, F^-, Br^-, SO_4^{2-}, NO_3^-, and PO_4^{3-}. The river water sample's total organic carbon (TOC) was determined using a Shimadzu TOC-L analyzer (Columbia, MD). The Hach spectrophotometer (DR 3900, Loveland, CO, USA) equipped with a TNT 828 kit was used for Total nitrogen (TN) analysis.

Text S10. PFAS analysis

Before quantifying PFAS, the samples obtained from the PFAS adsorption studies underwent centrifugation at a speed of 16,000 rpm for 15 min. Following the guidelines of EPA Method 537.1 Revision 2.0 (Zhang and Liang, 2022), the liquid portion of the sample was intentionally contaminated with $^{13}C_4$-PFOS and $^{13}C_2$-PFOA, which served as internal standards. The target PFASs in the prepared samples were quantified using a 1290 Infinity II LC system paired with a 6470 Triple Quad Mass Spectrometer (LC-MS/MS, Agilent Technologies, Santa Clara, CA, USA). The experiment used two Agilent Eclipse Plus C18 columns: a ZORBAX analytical column measuring 3 × 50 mm with a particle size of 1.8 μm and a delay column measuring 4.6 × 50 mm with a particle size of 3.5 μm. The columns were operated at a constant temperature of 50 °C. The binary mobile phase solvents A and B were selected as ammonium acetate (5 mM) in water and 95% methanol, respectively. The flow rates of these solvents were set at a constant value of 0.5 mL/min. The mobile phase gradient started with a composition of 70% A and 30% B, transitioned to a composition of 0% A and 100% B at 8 min, and maintained this composition for 4 min before returning to the original values. The whole duration was 12 min.

Table S1. Chemicals and reagents used in this study.

Chemicals and reagents	Grade/purity	Supplier details
Graphene oxide	15-20 sheets, 4-10% edge-oxidized	Sigma-Aldrich
Ascorbic acid	≥ 99%	Fisher Scientific
Ammonium hydroxide	28-30%	Fisher Scientific
Ethanolamine	≥ 99%	Fisher Scientific
Diethylene triamine	≥ 98%	Fisher Scientific
Hexamethylenetetramine	≥ 99%	Fisher Scientific
Poly(diallyldimethylammonium chloride)	35 wt.% in H_2O	Sigma-Aldrich
Cetyltrimethylammonium bromide	~ 98%	Fisher Scientific
Perfluorohexanoic acid	≥ 98%	Frontier Scientific
Perfluoroheptanoic acid	≥ 98%	Matrix Scientific
Perfluorooctanoic acid	≥ 96%	Sigma-Aldrich
Perfluorononanoic acid	≥ 98%	Oakwood Chemicals
Perfluorodecanoic acid	≥ 98%	Matrix Scientific
Perfluorobutanoic acid	≥ 98%	Matrix Scientific
Potassium perfluorobutanesulfonate	≥ 98%	Accela Chembio Inc
Perfluorohexanesulfonic acid potassium salt	≥ 98%	Frontier Scientific
Heptadecafluorooctanesulfonic acid potassium salt	≥ 98%	Sigma-Aldrich
Undecafluoro-2-methyl-3-oxahexanoic acid	≥ 97%	SynQuest Laboratories
6:2 Fluorotelomer sulfonic acid	≥ 98%	SynQuest Laboratories
Perfluoro-n-[1,2-$^{13}C_2$]octanoic acid	≥ 98%	Wellington Laboratories Inc.
Sodium perfluoro-1[1,2,3,4-$^{13}C_4$]octanesulfonate	≥ 98%	Wellington Laboratories Inc.
Perfluoro-n-[1,2,3,4,6-$^{13}C_5$]hexanoic acid	≥ 98%	Wellington Laboratories Inc.
Cetyltrimethyl ammonium chloride	≥ 95%	Tokyo Chemical Industry
Ammonium acetate	LC/MS Grade	Fisher Scientific
Methanol	LC/MS Grade	Fisher Scientific
Ethanol	99.5%	Fisher Scientific
Water	LC/MS Grade	Fisher Scientific

Table S2. The physicochemical properties of PFAS used in this study.

Category	Compound name	Chemical structure	Chemical formula	Molecular weight (g/mol)	S_w (25 °C) (g/L)	pK_a (25 °C)
Short-chain PFCA	Perfluorobutanoic acid (PFBA)	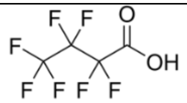	$C_4HF_7O_2$	214	2.14×10^{-3} (Registry, 2023)	0.08 (Registry, 2023)
Short-chain PFCA	Perfluorohexanoic acid (PFHxA)	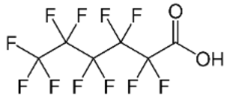	$C_6HF_{11}O_2$	314	15.7 (Fujii et al., 2007)	-0.16 (Steinle-Darling and Reinhard, 2008)
Short-chain PFCA	Perfluoroheptanoic acid (PFHpA)	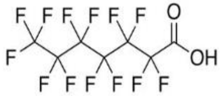	$C_7HF_{13}O_2$	364	3.65×10^{-3} (Kim et al., 2021)	−2.29 (Kim et al., 2021)
Long-chain PFCA	Perfluorooctanoic acid (PFOA)	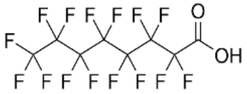	$C_8HF_{15}O_2$	414	3.4 (Fujii et al., 2007)	-0.2 (Steinle-Darling and Reinhard, 2008)
Long-chain PFCA	Perfluorononanoic acid (PFNA)	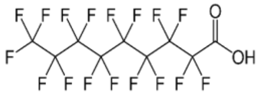	$C_9HF_{17}O_2$	464	6.25×10^{-2} (Kim et al., 2021)	-0.21 (Kim et al., 2021)
Long-chain PFCA	Perfluorodecanoic acid (PFDA)	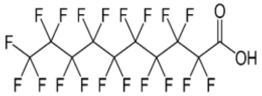	$C_{10}HF_{19}O_2$	514	9.5 (Christensen et al., 2022)	-5.2 (Pauletto and Bandosz, 2022)
Short-chain PFSA	Potassium perfluorobutanesulfonate (PFBS)	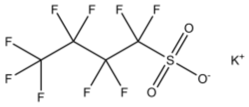	$C_4F_9O_3SK$	338	46.2 (Zhou et al., 2010)	0.14 (Steinle-Darling and Reinhard, 2008)

Long-chain PFSA	Perfluorohexanesulfonic acid potassium salt (PFHxS)	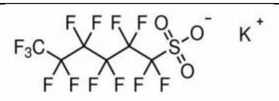	$C_6F_{13}KO_3SK$	438	2.3 (Christensen et al., 2022)	0.14 (Steinle-Darling and Reinhard, 2008)
Long-chain PFSA	Heptadecafluorooctanesulfonic acid potassium salt (PFOS)	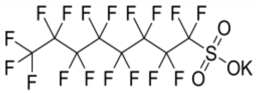	$C_8HF_{17}KO_3SK$	538	0.57 (Fujii et al., 2007)	-3.27 (Brooke et al., 2004)
PFOA alternative	Undecafluoro-2-methyl-3-oxahexanoic acid (GenX)	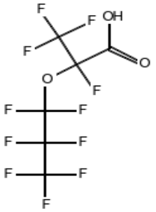	$C_6HF_{11}O_3$	330.05	N/A	2.84 (Pauletto and Bandosz, 2022)
PFOS alternative	6:2 fluorotelomer sulfonic acid (6:2 FTSA)	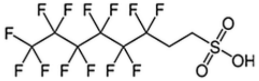	$C_8H_5F_{13}O_3S$	428	1.3 (Christensen et al., 2022)	1.31 (Christensen et al., 2022)

Note: Solubility in water (S_w); Dissociation constant (pK_a); N/A: Data not available

Table S3. Characteristics of the Hudson River water used in this study.

Parameter	Value
pH	7.37 ± 0.01
TN (mg/L)	1.00 ± 0.00
TOC (mg/L)	3.38 ± 0.07
Br^{-} (mg/L)	5.64 $\times 10^{-3}$ ± 0.00
PO_4^{3-} (mg/L)	7.60 $\times 10^{-3}$ ± 0.00
F^{-} (mg/L)	ND
Cl^{-} (mg/L)	ND
SO_4^{2-} (mg/L)	ND
NO_3^{-} (mg/L)	ND
PFBS (ng/L)	14.64 ± 0.23
PFHxA (ng/L)	25.50 ± 0.77
PFHxS (ng/L)	9.57 ± 0.83
6:2 FTSA (ng/L)	5.86 ± 0.77
PFNA (ng/L)	15.50 ± 3.15
PFDA (ng/L)	11.68 ± 1.98

Note: TN: total nitrogen; TOC: total organic carbon; ND: not detected.

Table S4. Dynamic multiple reaction monitoring transitions for the studied PFAS.

Compound name	Precursor ion	Product ion	Retention time (min)	Fragmentor	Collision Energy
PFBA	213	168.9	1.385	59	8
PFHxA	313	269	4.67	67	4
PFHxA	313	119	4.67	67	28
PFHpA	363	319	5.63	67	8
PFHpA	363	169	5.63	67	16
PFOA	413	369	6.3	61	8
PFOA	413	169	6.3	61	16
PFNA	463	419	6.8	67	16
PFNA	463	169	6.8	67	8
PFDA	513	469	7.24	73	20
PFDA	513	218.7	7.24	73	8
PFBS	299	99	3.6	121	32
PFBS	299	80	3.6	121	40
PFHxS	399	99	5.7	136	36
PFHxS	399	80	5.7	136	80
PFOS	499	99	6.84	159	76
PFOS	499	80	6.84	159	80
6:2 FTSA	427	406.8	6.56	122	24
6:2 FTSA	427	79.9	6.56	122	48
GenX	285	185	4.9	68	12
GenX	285	169	4.9	68	4
^{13}C-PFOA	415	370	6.3	61	8
^{13}C-PFOS	503	80	6.84	159	80

Table S5. Particle size, BET total specific surface area, and pore size distribution analysis were conducted at 100 °C**.**

Adsorbent	Particle size (μm)	Specific surface area (m^2/g)	Total pore volume (cm^3/g)	Maximum pore width (A°)
GO	2.54	262.75	0.26	204.74
GO-CTAC	0.67	14.23	0.02	204.61

Table S6. Summary of binding energy and atomic concentration of GO-CTAC.

Samples	Binding energy (eV)				
	C 1s	O 1s	N 1s	Cl 2p	F 1s
Before adsorption	284.6	532.5	401.3	198	-
After adsorption	284.8	532.3	399.6	-	688.7
	Atomic concentration (%)				
	C 1s	O 1s	N 1s	Cl 2p	F 1s
Before adsorption	87.58	10.58	1.45	0.39	-
After adsorption	77.45	8.69	2.69	0.07	11

Table S7. Adsorption performance comparison of the prepared GO-CTAC with other adsorbents reported in the literature.

Adsorbent	Experimental conditions	PFAS studied	Equilibrium time	Isotherm	Adsorption capacity (mg/g)	References
Activated carbon	Adsorbent dose = 250 mg/L, pH = 5, concentration = 0.5-250 mg/L	PFOA	240 min	Langmuir	157.1	Shaikh et al., 2023
CTAB-activated carbon					455.8	
Molecular imprinted polymer	Adsorbent dose = 200 mg/L, pH = 7, concentration = 0.005-1 mg/L	PFOA	600 min	Langmuir	5.45	Cao et al., 2016
Fe-doped graphitized biochar	Adsorbent dose = 1250 mg/L, pH = 6.5, concentration = 0-100 mg/L	PFOA, PFBA	2880 min	Langmuir	PFOA = 38.6<br>PFBA = 10.1	Liu et al., 2023
GAC					PFOA = 34.7<br>PFBA = 2.25	
PAC					PFOA = 37.5<br>PFBA = 3.89	
Fluorine doped mesoporous carbon	Adsorbent dose = 1000 mg/L, pH = 6.6, concentration = 1 mg/L	PFOS	7 d	N/A	0.99	Medha et al., 2024
PolyDADMAC-GAC	Adsorbent dose = 6666.66 mg/L, pH = N/A, concentration = 1 mg/L	PFBA, PFOA	5760 min	Langmuir	PFBA = 165<br>PFOA = 313	Ramos et al., 2022
GO-CTAC	Adsorbent dose = 100 mg/L, pH = 6.6, concentration = 0.01-0.5 mg/L	PFBA, PFBS, PFHxA, PFHxS, PFHpA, PFOA, PFOS, 6:2 FTSA, GenX, PFNA, PFDA	5 min	Sips	∑PFAS = 48.47	This study

Note: N/A: not available

Table S8. Parameters and values derived from adsorption isotherm models of Langmuir, Freundlich, Sips, and Toth for PFAS adsorption by GO-CTAC.

Model	Parameter	Value
Langmuir	R^2	0.986
	K_L (L/μg)	8.64 × 10^{-3}
	q_m (mg/g)	57.18
Freundlich	R^2	0.962
	K_F (mg·$L^{1/m}$/(g·$\mu g^{1/m}$))	2.52
	m	2.15
Sips	R^2	0.998
	K_S (L/μg)	1.38 × 10^{-2}
	q_m (mg/g)	48.47
	n	0.58
Toth	R^2	0.993
	K_T (L/μg)	7.58 × 10^{-3}
	q_m (mg/g)	43.86
	t	4.19

Table S9. Experimental and isotherm modeled values of total adsorbed PFAS at equilibrium (q_e) and concentrations in aqueous phase at equilibrium (C_e) in the adsorption process by GO-CTAC.

∑PFAS C_e (ppb)	∑PFAS q_e experimental (mg/g)	∑PFAS q_e modeled (mg/g)			
		Langmuir	Freundlich	Sips	Toth
1.26	1.27	0.61	2.80	0.05	0.42
1.14	2.30	0.56	2.68	0.04	0.38
21.69	5.34	9.03	10.56	5.45	7.21
70.98	23.91	21.74	18.33	23.88	23.19
560.57	47.07	47.40	47.95	47.08	43.84

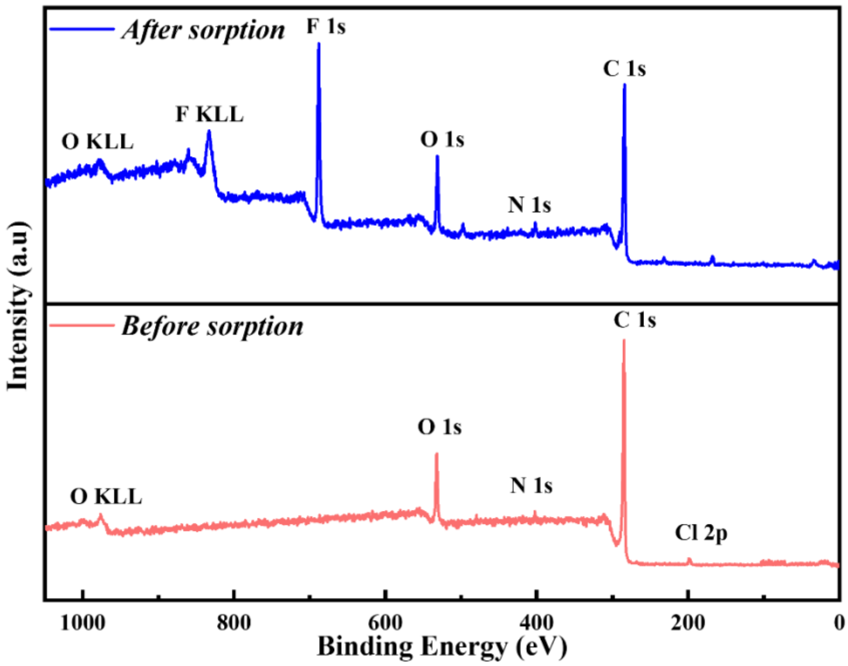

Figure S1. XPS survey of GO-CTAC (before adsorption) and GO-CTAC (after adsorption).

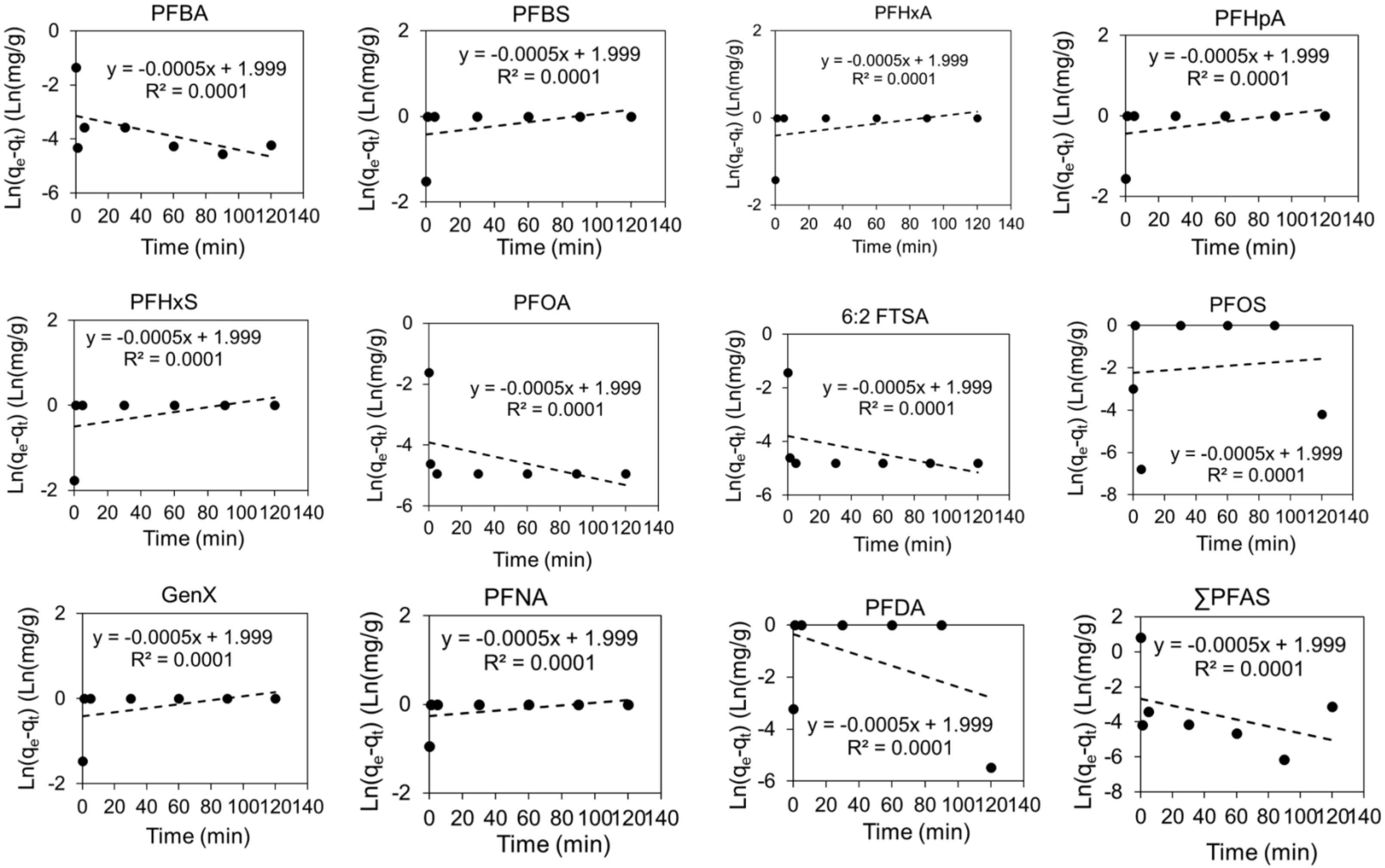

Figure S2. Fitting of adsorption data of PFAS at the initial concentrations of 20 µg/L by the linear form of pseudo-first-order model.

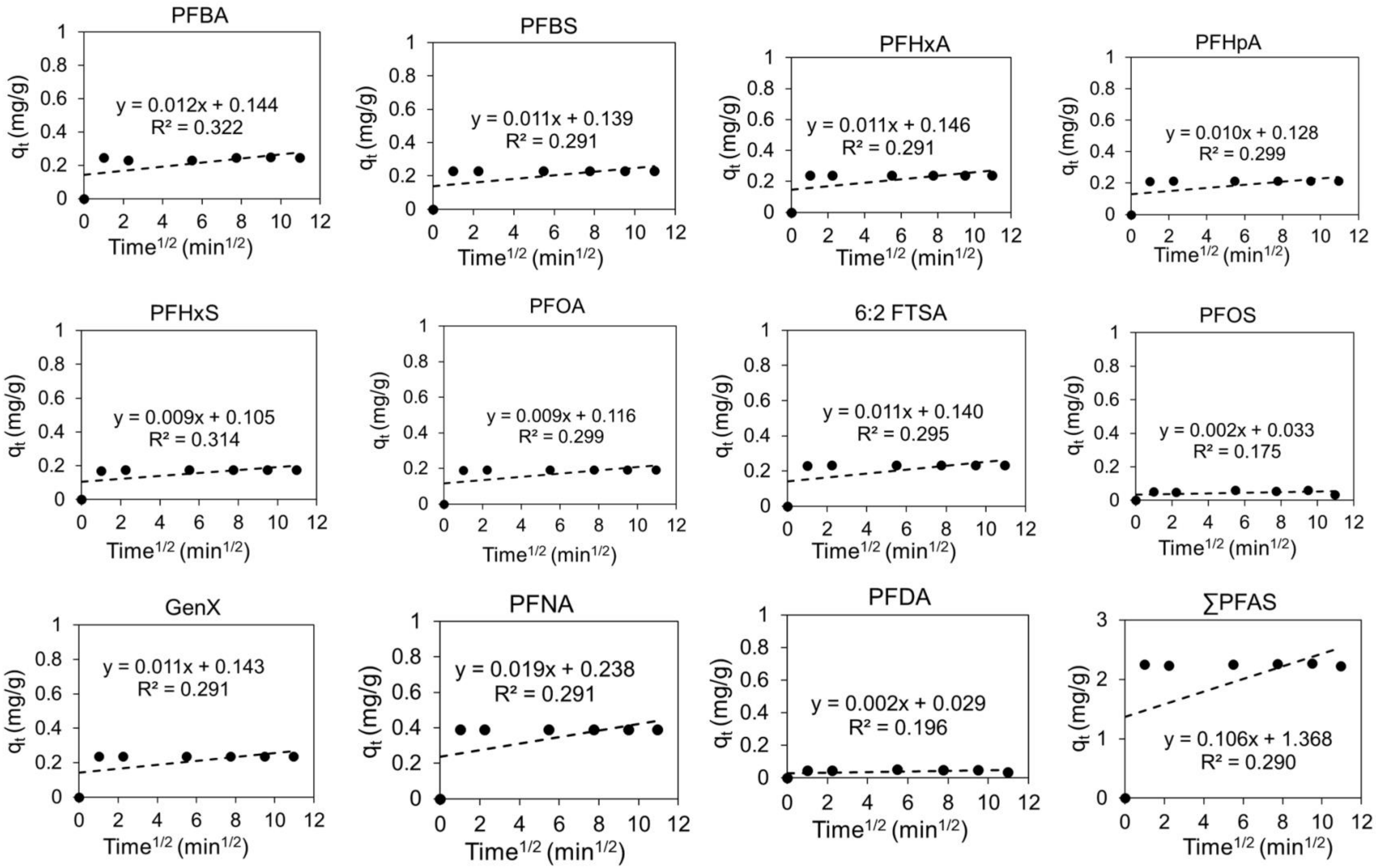

Figure S3. Fitting of adsorption data of PFAS at the initial concentrations of 20 μg/L by the linear form of intra-particle diffusion model.

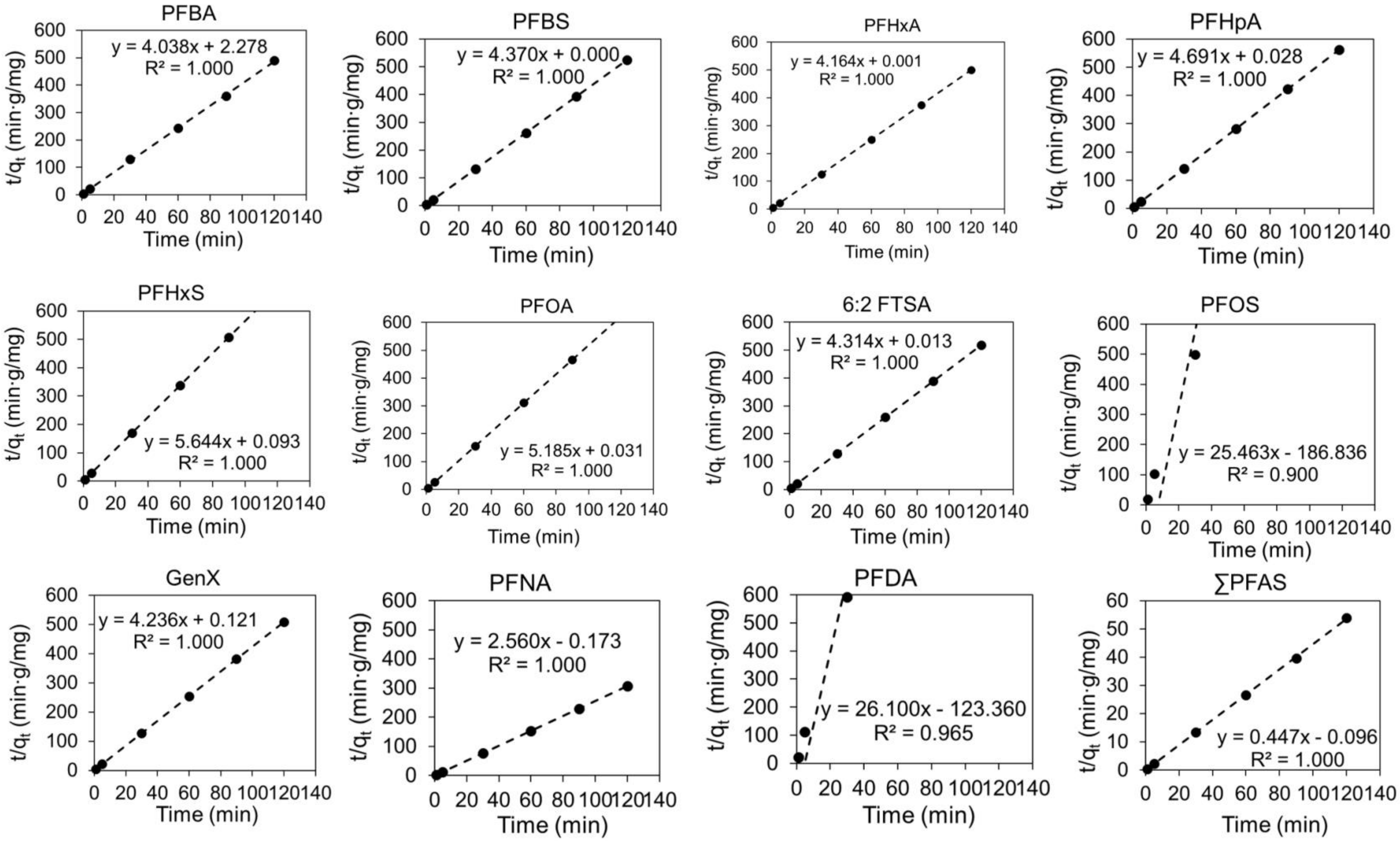

Figure S4. Fitting of adsorption data of PFAS at the initial concentrations of 20 μg/L by the linear form of pseudo-second-order model.

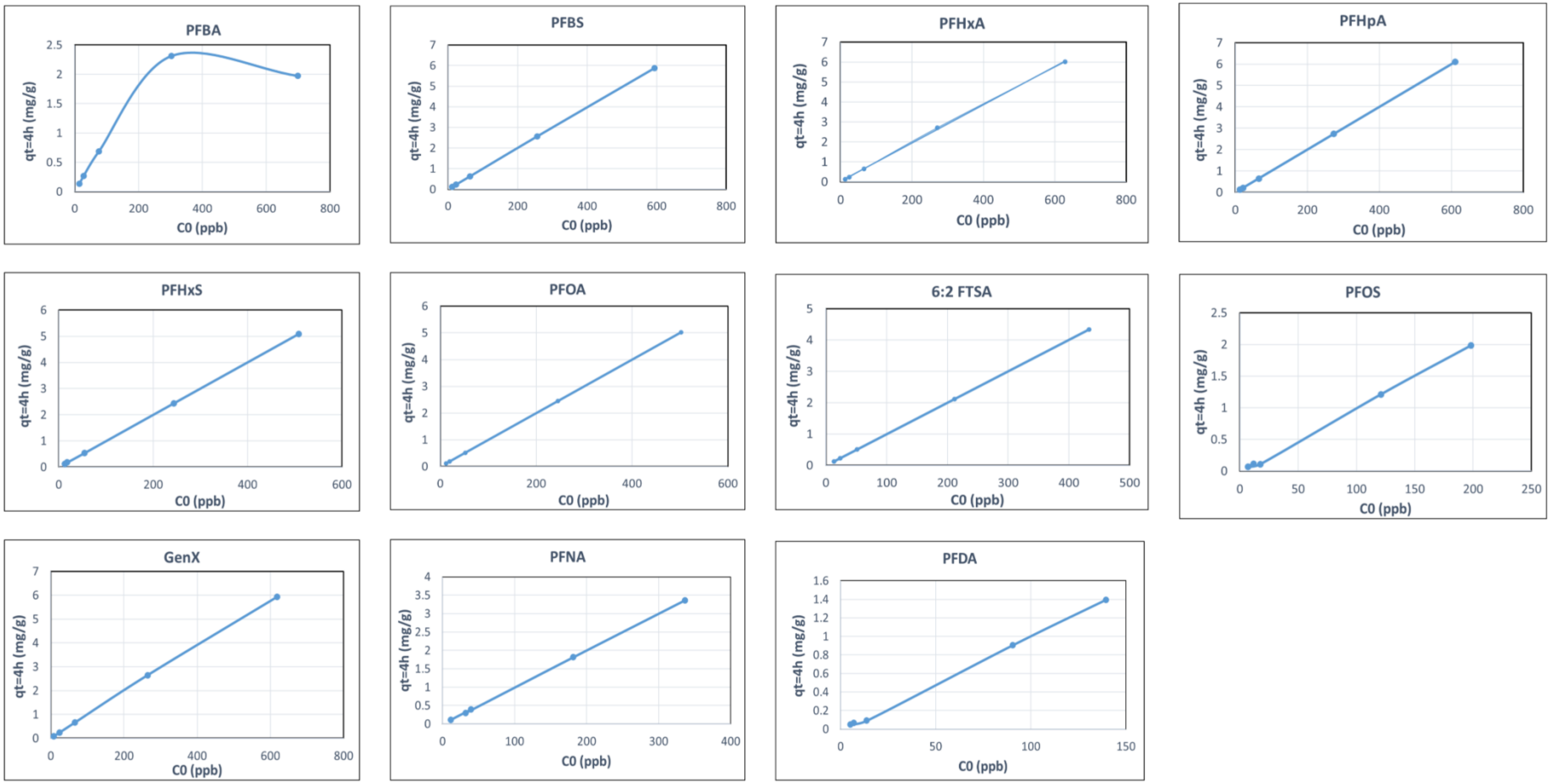

Figure S5. The relationship between initial concentrations (C_0) and mass of PFAS adsorbed by the GO-CTAC.

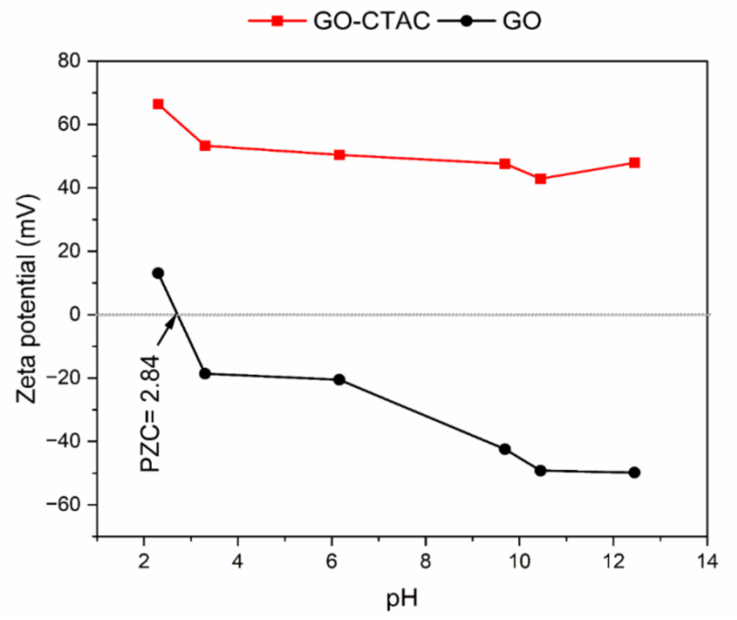

Figure S6. Zeta potential of the GO and GO-CTAC, respectively.

References

Brooke, D., Footitt, A., Nwaogu, T., 2004. Environmental risk evaluation report: Perfluorooctanesulphonate (PFOS). Environment Agency, Bristol, United Kingdom.

Cao, F., Wang, L., Ren, X., Sun, H., 2016. Synthesis of a perfluorooctanoic acid molecularly imprinted polymer for the selective removal of perfluorooctanoic acid in an aqueous environment. Journal of Applied Polymer Science 133 (15), 43192.

Chen, X., Li, Y., Yuan, X., Li, N., He, W., Liu, J., 2020. Synergistic effect between poly(diallyldimethylammonium chloride) and reduced graphene oxide for high electrochemically active biofilm in microbial fuel cell. Electrochimica Acta 359, 136949.

Christensen, E.R., Wang, Y., Huo, J., Li, A., 2022. Properties and fate and transport of persistent and mobile polar organic water pollutants: A review. Journal of Environmental Chemical Engineering 10 (2), 107201.

De Silva, K.K.H., Huang, H.-H., Yoshimura, M., 2018. Progress of reduction of graphene oxide by ascorbic acid. Applied Surface Science 447, 338-346.

Fernández-Merino, M.J., Guardia, L., Paredes, J.I., Villar-Rodil, S., Solís-Fernández, P., Martínez-Alonso, A., Tascón, J.M.D., 2010. Vitamin C Is an Ideal Substitute for Hydrazine in the Reduction of Graphene Oxide Suspensions. The Journal of Physical Chemistry C 114 (14), 6426-6432.

Fujii, S., Polprasert, C., Tanaka, S., Hong Lien, N.P., Qiu, Y., 2007. New POPs in the water environment: distribution, bioaccumulation and treatment of perfluorinated compounds – a review paper. Journal of Water Supply: Research and Technology – AQUA 56 (5), 313-326.

Jiang, L., Hu, X., Yang, B., Yang, Z., Lu, C., 2023. Preparation of porous diethylene triamine reduced graphene oxide aerogel for efficient pollutant dye adsorption. Journal of Porous Materials 30 (5), 1485-1497.

Jiang, T., Pervez, M.N., Quianes, M.M., Zhang, W., Naddeo, V., Liang, Y., 2023. Effective stabilization of per-and polyfluoroalkyl substances (PFAS) precursors in wastewater treatment sludge by surfactant-modified clay. Chemosphere 341, 140081.

Jiang, T., Zhang, W., Liang, Y., 2022. Uptake of individual and mixed per-and polyfluoroalkyl substances (PFAS) by soybean and their effects on functional genes related to nitrification, denitrification, and nitrogen fixation. Science of The Total Environment 838, 156640.

Kim, S., Chen, J., Cheng, T., Gindulyte, A., He, J., He, S., Li, Q., Shoemaker, B.A., Thiessen, P.A., Yu, B., Zaslavsky, L., Zhang, J., Bolton, E.E., 2021. PubChem in 2021: new data content and improved web interfaces. Nucleic Acids Research 49 (D1), D1388-D1395.

Lee, J.W., Ko, J.M., Kim, J.-D., 2012. Hydrothermal preparation of nitrogen-doped graphene sheets via hexamethylenetetramine for application as supercapacitor electrodes. Electrochimica Acta 85, 459-466.

Liu, Z., Zhang, P., Wei, Z., Xiao, F., Liu, S., Guo, H., Qu, C., Xiong, J., Sun, H., Tan, W., 2023. Porous Fe-doped graphitized biochar: An innovative approach for co-removing per-/polyfluoroalkyl substances with different chain lengths from natural waters and wastewater. Chemical Engineering Journal 476, 146888.

Ma, C., Qian, Y., Zhang, S., Song, H., Gao, J., Wang, S., Liu, M., Xie, K., Zhang, X., 2018. Temperature-controlled ethanolamine and Ag-nanoparticle dual-functionalization of graphene oxide for enhanced electrochemical nitrite determination. Sensors and Actuators B: Chemical 274, 441-450.

Medha, S., Romisher, Z., Van Bramer, S., Weyrich, J., Khan, S., Saha, D., 2024. Enhanced adsorption of perfluorooctanesulfonic acid (PFOS) in fluorine doped mesoporous carbon: Experiment and simulation. Carbon 218, 118745.
Pauletto, P.S., Bandosz, T.J., 2022. Activated carbon versus metal-organic frameworks: A review of their PFAS adsorption performance. Journal of Hazardous Materials 425, 127810.
Ramos, P., Singh Kalra, S., Johnson, N.W., Khor, C.M., Borthakur, A., Cranmer, B., Dooley, G., Mohanty, S.K., Jassby, D., Blotevogel, J., Mahendra, S., 2022. Enhanced removal of per- and polyfluoroalkyl substances in complex matrices by polyDADMAC-coated regenerable granular activated carbon. Environmental Pollution 294, 118603.
Agency for Toxic Substances and Disease Registry, 2023. Chemical and physical information. https://www.atsdr.cdc.gov/toxprofiles/tp17-c4.pdf.
Shaikh, M.A.N., Sarkar, P., Nawaz, T., 2023. PFOA remediation from aqueous media using CTAB impregnated activated carbon: A closed-loop sustainable study with comprehensive selectivity analysis. Journal of Water Process Engineering 54, 103965.
Steinle-Darling, E., Reinhard, M., 2008. Nanofiltration for trace organic contaminant removal: structure, solution, and membrane fouling effects on the rejection of perfluorochemicals. Environmental Science & Technology 42 (14), 5292-5297.
Yang, Y.J., Li, W., 2014. CTAB functionalized graphene oxide/multiwalled carbon nanotube composite modified electrode for the simultaneous determination of ascorbic acid, dopamine, uric acid and nitrite. Biosensors and Bioelectronics 56, 300-306.
Zhang, W., Jiang, T., Liang, Y., 2022a. Stabilization of per-and polyfluoroalkyl substances (PFAS) in sewage sludge using different sorbents. Journal of Hazardous Materials Advances 6, 100089.
Zhang, W., Liang, Y., 2022. Performance of different sorbents toward stabilizing per-and polyfluoroalkyl substances (PFAS) in soil. Environmental Advances 8, 100217.
Zhang, W., Zhang, Q., Liang, Y., 2022b. Ineffectiveness of ultrasound at low frequency for treating per-and polyfluoroalkyl substances in sewage sludge. Chemosphere 286, 131748.
Zhou, Q., Deng, S., Yu, Q., Zhang, Q., Yu, G., Huang, J., He, H., 2010. Sorption of perfluorooctane sulfonate on organo-montmorillonites. Chemosphere 78 (6), 688-694.
